# Chromatin dynamics and the transcriptional competence of HSV-1 genomes during lytic infections

**DOI:** 10.1371/journal.ppat.1008076

**Published:** 2019-11-14

**Authors:** MiYao Hu, Daniel P. Depledge, Esteban Flores Cortes, Judith Breuer, Luis M. Schang

**Affiliations:** 1 University of Alberta, Department of Biochemistry, Edmonton, AB, Canada; 2 Cornell University, College of Veterinary Medicine, Baker institute for animal health, Ithaca, NY, United States of America; 3 6401Division of Infection and Immunity, University College London, London, United Kingdom; LSU Eye Center Department of Ophthalmology, UNITED STATES

## Abstract

During latent infections with herpes simplex virus 1 (HSV-1), viral transcription is restricted and the genomes are mostly maintained in silenced chromatin, whereas in lytically infected cells all viral genes are transcribed and the genomes are dynamically chromatinized. Histones in the viral chromatin bear markers of silenced chromatin at early times in lytic infection or of active transcription at later times. The virion protein VP16 activates transcription of the immediate-early (IE) genes by recruiting transcription activators and chromatin remodelers to their promoters. Two IE proteins, ICP0 and ICP4 which modulate chromatin epigenetics, then activate transcription of early and late genes. Although chromatin is involved in the mechanism of activation of HSV- transcription, its precise role is not entirely understood. In the cellular genome, chromatin dynamics often modulate transcription competence whereas promoter-specific transcription factors determine transcription activity. Here, biophysical fractionation of serially digested HSV-1 chromatin followed by short-read deep sequencing indicates that nuclear HSV-1 DNA has different biophysical properties than protein-free or encapsidated HSV-1 DNA. The entire HSV-1 genomes in infected cells were equally accessible. The accessibility of transcribed or non-transcribed genes under any given condition did not differ, and each gene was entirely sampled in both the most and least accessible chromatin. However, HSV-1 genomes fractionated differently under conditions of generalized or restricted transcription. Approximately 1/3 of the HSV-1 DNA including fully sampled genes resolved to the most accessible chromatin when HSV-1 transcription was active, but such enrichment was reduced to only 3% under conditions of restricted HSV-1 transcription. Short sequences of restricted accessibility separated genes with different transcription levels. Chromatin dynamics thus provide a first level of regulation on HSV-1 transcription, dictating the transcriptional competency of the genomes during lytic infections, whereas the transcription of individual genes is then most likely activated by specific transcription factors. Moreover, genes transcribed to different levels are separated by short sequences with limited accessibility.

## Author summary

Although chromatin epigenetics modulate transcription of the nuclear replicating DNA viruses, and play major roles in the process of establishment of, and reactivation from, latency, the specific mechanisms of this modulation are not totally clear. Chromatin often regulates the transcriptional competency of cellular genes, rather than the actual level of transcription of individual genes. Here, we show that chromatin dynamics regulate the transcription competency of entire herpes simplex virus 1 (HSV-1) genomes, rather than the actual transcription level of individual genes. Moreover, CTCF/ insulator containing sequences flanking the immediate-early gene loci are more inaccessible when these genes are highly transcribed in a context of little transcription from the rest of the genome than when no gene was highly transcribed or all genes were. We postulate that chromatin dynamics modulate the transcriptional competency of the HSV-1 genome. Genes in genomes rendered transcriptionally inactive by chromatin dynamics cannot be transcribed, whereas transcription of individual genes, or of group of genes, is regulated separately in the transcriptionally competent genomes.

## Introduction

Herpes simplex virus 1 (HSV-1) is a double stranded nuclear replicating DNA virus. It establishes latent infections, in which viral transcription is restricted, and lytic infections, in which all viral genes are transcribed. The HSV-1 DNA is maintained in silenced chromatin during latency [1–4] but is in most dynamic chromatin during lytic infections [5–7]. Consequently, micrococcal nuclease (MCN) cleaves HSV-1 DNA into mono- to poly-nucleosome sized fragments in latent infection [1], or to heterogeneously sized fragments in lytic infections [5, 8–11]. A subset of the latter fragments resolve together with cellular mono- or di-nucleosomes during differential centrifugations [5,6], and associates with all core histones [12]. Complete MCN digestion has suggested that several regions of the HSV-1 genome could be nucleosome-free [13], whereas “serial” MCN protection assays indicate that HSV-1 DNA in lytic infections is chromatinized in highly dynamic nucleosomes [5, 6]. HSV-1 DNA may also associate preferentially with ICP4 rather than with histones [14, 15].

Transcription of the five HSV-1 immediate early (IE) genes is activated by the tegument protein VP16 immediately after the HSV-1 genomes enter the nucleus. VP16 interacts with the host cell factor 1 (HCF-1) and octamer-binding transcription factor 1 (Oct-1) to be recruited to the TAATGARAT motifs on the promoters of HSV-1 IE genes[16–18]. This complex then recruits chromatin remodelers to allow IE gene transcription [16, 19–26]. Transcription of the early genes (E) is activated by the IE proteins ICP4, ICP0 and ICP22. ICP4, which is essential for the activation of transcription of E and L genes [27], recruits chromatin remodelers to the HSV-1 genomes [28, 29] and induces histone dynamics [7]. ICP0, which enhances IE, E, and L gene expression, induces the degradation of silencing histone variants and affects apparent histone occupancy [30–34]. DNA replication occurs after E proteins are expressed, and late genes (L) are transcribed after the onset of HSV-1 DNA replication. The mechanisms preventing E and L gene transcription before IE proteins are expressed or DNA replication starts, respectively, remain only partially understood [35, 36].

Via many mechanisms such as recruitment of transcription factors or heterochromatin proteins and nucleosome stability, chromatin normally regulates nuclear DNA accessibility and, consequently, DNA-dependent processes such as transcription, replication or DNA repair [37–40]. HSV-1 DNA associates with histones bearing markers of silencing early in infection or with markers of transcription at later times [11, 19, 32, 41–43]. Inhibition of certain histone demethylases modulates HSV-1 transcription [44–47]. Histone acetyltransferases (HATs) such as GCN5, PCAF, and CBP/p300 are recruited to HSV-1 chromatin by VP16 [16, 19, 48], although knockdown of these HATs has limited effects on HSV-1 transcription [49]. Inhibition of histone deacetylases (HDACs), such as CLOCK, SWI/SNF and REST/Co-REST complexes, increases HSV-1 transcription or replication [29, 50–53]. Chromatin thus performs important roles in the regulation of HSV-1 transcription, and epigenetic modifications affect HSV-1 transcription and replication. However, the precise mechanisms of HSV-1 transcription regulation by chromatin remain incompletely understood.

Chromatin often regulates cellular transcription competence. DNA chromatinized to silenced states, often via the recruitment of silencing proteins to specific histone modifications and exclusion of transcription proteins, is not accessible to transcription complexes. With the notable exception of binding by the pioneering factors, the promoter sequences in this chromatin often become accessible to transcription factors only after the chromatin is remodelled [54, 55]. Binding of the promoter-specific transcription factors to the now accessible promoters then determines the transcription activity of each specific gene.

Here, we explored the dynamics of HSV-1 chromatin in lytically infected cells by biophysical fractionation of serially digested HSV-1 chromatin and short-read deep sequencing analyses of each fraction. Within the constraints of genome-wide population analyses, which preclude the observation of changes in minority subsets of genomes, HSV-1 genomes were dynamically chromatinized as units and the global dynamics of the viral chromatin were related to transcription activity. The subpopulation of the viral genomes in the most dynamic chromatin including fully sampled genes was enriched under conditions of active transcription but depleted when transcription was restricted. Short sequences with limited accessibility separated loci with different transcription levels.

## Materials and methods

### Cells and virus

Vero African green monkey kidney fibroblast cells (originally purchased from ATCC and maintained in the Schang lab since 2000) were maintained in Dulbecco's modified minimum Eagle's medium (DMEM) supplemented with 5% fetal bovine serum (FBS). Low-passage (p11 to 15) HSV-1 strain KOS was used throughout this study. Viral stocks were propagated and titrated on monolayers of Vero cells as previously described [5, 6, 56]. Vero cells were infected with 10 plaque forming unit (pfu) per cell in serum-free medium as previously described [5, 6, 56], with modifications. Briefly, Vero cells were inoculated for 1 h at 37°C, inocula were then removed, and the cells were washed twice with 4°C phosphate-buffered saline (PBS– 1 mM KH_2_PO_4_, 154 mM NaCl, 3 mM Na_2_HPO_4_ [pH 7.4]) or SFM. Fresh pre-warmed DMEM-5% FBS was added to the infected cells, which were then harvested at 7 hours post-infection (hpi) unless otherwise stated.

### Drugs

Cycloheximide (CHX) 5 mg/ml stock solution was prepared in serum free medium (SFM) and diluted to 50 μg/ml in DMEM-5% FBS, SFM or PBS before use. Cells were pre-treated with 50 μg/ml CHX in DMEM-5% FBS for 1 h prior to infection. CHX was maintained in the inocula, PBS or SFM washes and DMEM-5% FBS until harvest.

Phosphonoacetic acid (PAA) 100 mM stock solution was prepared in SFM and adjusted to pH 7.0 with 1 N NaOH. The stock was diluted to 400 μM in DMEM-5% FBS and added to the cells after removing inocula and washing with PBS.

Roscovitine (Rosco) 100 mM stock solution was prepared in dimethyl sulfoxide (DMSO). The stock was diluted to 100 μM in pre-warmed DMEM-5% FBS and added to the cells after removing the inocula and washing with PBS or SFM.

### Chromatin extraction

Chromatin of Vero cells was extracted as described [5, 6], with minor modifications. Briefly, each 1 x 10^7^ cells were washed with 4°C PBS, trypsinized, and resuspended in 15 ml of DMEM-5% FBS. Cells were then pelleted and resuspended in 15 ml 4°C PBS. Cells were pelleted again, and resuspended in 15 ml of 4°C reticulocyte standard buffer (RSB, 10 mM Tris [pH 7.5], 10 mM NaCl, 5 mM MgCl_2_). Cells were pelleted again, resuspended in 10 ml 4°C RSB and incubated on ice for 10 min. The cell membrane was lysed by adding 0.5 ml 10% (vol/vol) triton X-100 to every 10 ml of cells in RSB and incubating on ice for 10 min, flipping the tubes upside down thrice. Nuclei were then pelleted by centrifugation at 3,250 xg for 25 min at 4°C.

Each 1 x 10^7^ nuclei were resuspended and lysed in 1.5 ml chromatin extraction buffer (1 mM ethylene glycol-bis(beta-aminoethyl ether)-N, N, N’, N’,- tetra-acetic acid (EGTA), 2% Triton X-100, 3 mM MgCl_2_, 2 mM Tris [pH 8]) on ice for 30 min. Every 5 min, nuclei were mixed by pipetting up and down 10 times using a P1000 at a setting of 750 μl. Chromatin was spun down at 2,000 xg for 10 min at 4°C, and resuspended in 200 μl per 1 x 10^7^ cells MCN digestion buffer (10 mM Tris [pH 8], 1 mM CaCl_2_).

### Nuclease protection assay

Serial MCN digestion was performed basically as described [5, 6]. Briefly, 5% of the chromatin suspension was taken as unfractionated and undigested chromatin, directly subjected to proteinase K digestion (1mg/ml proteinase K with 0.5% SDS) overnight at 37°C and purified by phenol: chloroform extraction and ethanol precipitation. The DNA purified from this no-MCN digested chromatin was then sonicated into smaller fragments prior to resolution in agarose gels or sequencing. The remaining chromatin was pelleted at 2,000 xg for 5 min. The chromatin pellet was disrupted by racking on a rack, resuspended in 40 μl of MCN digestion buffer with 0.05 U MCN per each 1 x 10^7^ cells, and immediately spun down at 800 xg for 5 min. The supernatant (soluble chromatin) was removed and the MCN was immediately quenched with 10 μl of 0.05 M EGTA to prevent further digestion of the DNA released in the soluble chromatin. The pellet (insoluble chromatin) was disrupted as before and resuspended with fresh 40 μl of MCN digestion buffer with 0.05 U MCN per each 1 x 10^7^ nuclei. The entire procedure was repeated six times. The supernatants from the six rounds of digestion were pooled and resolved together in sucrose gradients.

### Encapsidated or protein-free HSV-1 DNA

Vero cells were infected with HSV-1 at a multiplicity of infection (moi) of 5. After inoculation for 1 h at 37°C, the inoculum was removed, the cells were washed and fresh DMEM-5% FBS was added to the infected cells. The supernatant was collected after 24 h, to minimize contamination with cellular DNA from lysed cells. Virions were spun down at 10,000 xg for 2 h, resuspended in serum free media (SFM), and titrated. HSV-1 membranes were lysed with 0.5% Triton X-100 on ice for 10 min by pipetting up and down using P1000 at the setting of 500 μl. The capsids were then spun down at 15,000 xg for 1 h.

For deproteinized HSV-1 DNA, an aliquot of the purified HSV-1 capsids were subject to proteinase K digestion (10 mg/ml proteinase K, 1% SDS) overnight at 37°C. Deproteinized HSV-1 DNA was purified by phenol-chloroform extraction and ethanol precipitation, and then resuspended in TE (10 mM Tris (pH8.0), 1 mM ethylenediaminetetraacetic acid (EDTA) buffer.

### Sucrose gradient ultracentrifugation

Continuous 0 to 10% sucrose gradients were prepared using a Gradient Master instrument with sucrose gradient buffer (SGB) (10 mM Tris [pH 8.0], 1.5 mM MgCl_2_, and 0.5 M EGTA) containing 200 mM NaCl. Soluble chromatin was loaded on top of the gradients and centrifuged at 200,000 x*g* in a SW-40 Ti rotor for 180 min at 4°C. Approximately 1 ml fractions were then collected from the bottom of the tube. The pellet was recovered in 1 ml of STE (10 mM Tris [pH 7.5], 100 mM NaCl, 1 mM EDTA) after the removal of the final fraction.

To analyze the DNA, each fraction was subjected to proteinase K digestion (1mg/ml, 0.5% SDS) at 37°C overnight. The DNA was extracted by phenol-chloroform, precipitated with isopropanol, washed twice with 70% ethanol, and resuspended in TE (10mM Tris, 1mM EDTA, pH8.0) buffer. Ten percent of the DNA in each fraction was resolved in 2% agarose gels and visualized by incubating in 0.1mg/ml EtBr for 1h. The remaining DNA was sealed and shipped on ice packs for sequencing.

To analyze proteins, each chromatin fraction was subjected to trichloroacetic acid (TCA) precipitation, to a final concentration of 25% (V / V), incubated on ice for 30 min. Fractions were spun down at 20,000 xg for 30 min at 4°C, and the pellet was washed twice with -20°C acetone. The final protein pellet was resuspended in loading buffer (2% SDS, 10% glycerol, 60 mM Tris (pH 7.4), 0.01% bromophenol blue, 10 mM DTT), and denatured at 100°C for 10 min. Proteins were resolved in 14% SDS gels for resolution of histones, or 10% SDS gels for resolution of ICP4 or ICP8, at 15 V for 1 h and then 30 V until the front dye ran out of the gel.

### Hybridization

After visualization, agarose gels were soaked in 1 M HCl for 30 min on a shaker at room temperature, rinsed with double distilled water, and soaked in alkaline transfer buffer (0.4 N NaOH, 1 M NaCl) for 15 min at room temperature on a shaker. The alkaline transfer buffer was replaced for another 15 min at room temperature. Gels were rinsed with double distilled water, and soaked in neutralizing buffer (1 M Tris (pH7.4), 1.5 M NaCl) for 15 min at room temperature on a shaker. BioDyne B0.45 nylon membranes were cut to size and soaked in 10x SSC buffer (1.5 M NaCl, 150 mM trisodium citrate, pH 7.0). DNA in the agarose gels was capillary transferred according to standard protocols. Membranes were prehybridized with 10 ml rapid hybrid buffer (Amersham Biosciences, Piscataway, NJ) for one hour at 75°C in a rotating oven. The probe (HSV-1 EcoRI restriction fragment JK, [5, 6]) was labeled by random priming according to the manufacturer's instructions (Amersham Biosciences). The denatured probe was added to 5 ml of rapid hybrid buffer pre-warmed at 75°C, and hybridized in the rotating oven for 4 h at 75°C. Membranes were washed twice for 15 min with hybridization wash buffer 1 (75 mM NaCl, 7.5 mM sodium citrate, 0.5% SDS) at room temperature, rinsed with distilled water, and exposed to Kodak PhosphorImager screens. Signal was quantitated in a Bio-Rad FX molecular imager.

### Western blot

Gels were soaked in transfer buffer (25 mM Tris, 190 mM glycine) for 30 min. PVDF membranes were cut to size, activated in 100% methanol for 5 min, and soaked in transfer buffer for 20 min. Proteins were transferred to the PVDF membranes in a TransBlot Turbo transfer system (Bio-Rad) at 1 A for 30 min. PVDF membranes were air dried and re-activated with 100% methanol for 2 min prior to blocking. Membranes were blocked in 10 ml Odyssey blocking buffer (Cat. 427–40000) for 1 h at room temperature with shaking. Antibodies against histone H2A (Abcam, rabbit, Cat. ab88770), H2B (Abcam, mouse, Cat. ab52484), H3 (Abcam, rabbit, Cat. ab1791), H4 (Abcam, mouse, Cat. ab31830) were diluted 1:1000 in 10 ml PBS with 0.05% tween-20. Antibodies against ICP4 (Virusys, mouse, Cat. P1101), VP5 (Virusys, mouse, Cat. HA018) and ICP8 (Abcam, mouse, Cat. ab20194) were diluted 1:500, 1:250, or 1:50, respectively, in the same buffer. Membranes were probed for 2 h at room temperature for histones, or for 18 h at 4°C for ICP4, VP5 and ICP8, and then washed twice for 15 min each with 10 ml PBS at room temperature. Membranes were incubated with goat anti-mouse (680LT), goat anti-mouse (Alexa 488), or anti-rabbit (800LT) secondary antibodies diluted 1:1000 in 10 ml PBS, 0.05% tween-20 for 1 h at room temperature. Membranes were washed once with 10 ml PBS, 0.05% tween-20 for 15 min, and once with 10 ml PBS for 10 min. Secondary antibodies were visualized in a Li-Cor Odyssey system.

### Chromatin Immunoprecipitation

The samples containing soluble, insoluble, and undigested chromatin where adjusted to 540 μl with MCN-digestion buffer (10 mM Tris [pH 8], 1 mM CaCl_2_) and cross-linked by adding 135 μl of buffered 5% formaldehyde solution (5% CH_2_O, 100 mM Na_2_HPO_4_ pH 8.67), to a final concentration of 1%, for 10 min at 4°C with constant mixing. Cross-linking was quenched with 45 μl of 2 M glycine, to a final concentration of 125 mM, for 5 min at room temperature. The soluble chromatin was subjected to sucrose gradient fractionation, whereas the insoluble and undigested chromatin was centrifuged at 4,000 xg for 10 min at 4°C, resuspended with 1 ml of chromatin immunoprecipitation binding buffer and kept on ice. Fractionation was analysed by gel electrophoresis (5% of each fraction).

The insoluble chromatin, the undigested chromatin, and the chromatin fractions were dialyzed against ChIP binding buffer (1% Triton X-100, 10 mM Tris pH 8.0, 150 mM NaCl, 2 mM EDTA pH 8.0) overnight at 4°C. The insoluble chromatin, the undigested chromatin and fractions 1–6, which contained chromatin fragments longer than 1,500 bp, were sheared by sonication. Histone H3 antibody (ab1791; 0.375 μg) or nonspecific IgG (ab171870; 0. 375 μg) per ChIP reaction were conjugated to 56 μg of Dyna Beads (Invitrogen 10001D) for ~20 hrs at 4°C with constant rotation. After setting aside 2% to evaluate the input DNA, equal amounts of each fraction were incubated with H3 or IgG bead-ab complexes for ~25 hrs at 4°C with constant rotation. Immunopurified complexes were rinsed consecutively with 1 ml of washing solution 1 (1% Triton, 2 mM EDTA, 20 mM Tris pH 8.0, 150 mM NaCl), 2 (2 mM EDTA, 20 mM Tris pH 8.0, 150 mM NaCl), 3 (1% Triton, 2 mM EDTA, 20 mM Tris pH 8.0, 500 mM NaCl), 4 (2 mM EDTA, 20 mM Tris pH 8.0, 500 mM NaCl), 5 (1% NP-40, 1% NaDOC, 1 mM EDTA, 10 mM Tris pH 8.0, 250 mM LiCl), and 6 (1 mM EDTA, 10 mM Tris pH 8.0, 250 mM LiCl). Immunopurified complexes were eluted with 600 μl of ChIP elution buffer (10 mM Tris-HCl pH 8.0, 150 mM NaCl, 1 mM EDTA, 0.5% SDS) and 6 μl of proteinase K (20 mg/ml), and then incubated at 65°C for ~19 hrs to decrosslink and deproteinize. DNA was extracted using phenol-chloroform, isopropanol precipitated, ethanol washed, and subjected to saturating PCR (Fast SYBR Green master mix) with 300 nM each the of UL25 (5’-AGCCGTCCCTTCGGAGGCCA-3’, 5’-TCGGCCCGGTACAGCGGAAGCAC-3’), gE (5’-CCACCCCCGACCCCAGCCGA-3’, 5’-GGGGTCCCGCTGGCGGGAGT-3’), ICP4 (5’-CTGTCGCGACGAGACGGCGT-3’, 5’-GCCCCCGCGGCGGGCACCGA-3’) or GAPDH (5’- CCTCCTGCACCACCAACTGCTT -3’, 5’- GTCCCATTCCCCAGCTCTCATACC -3’) primers, or 75 nM each of the UL46 (5’-TCCTCGTAGACACGCCCCCCGT-3’, 5’-ACGCCCCCTACGAGGACGACGAGT -3’) primers. Purified DNA from the insoluble chromatin and the undigested chromatin were diluted 1:10 before PCR. PCR conditions: 20 sec denaturation at 95°C followed by 28 cycles of 3 sec denaturation at 95°C and 30 sec annealing/elongation at 60°C, followed by a final 5 min elongation at 65°C (all viral primers), or 20 sec denaturation at 95°C, followed by 35 cycles of 3 sec denaturation at 95°C and 30 sec annealing/elongation at 60°C, followed by a final 5 min elongation at 65°C (GAPDH). PCR products were analysed by gel electrophoresis.

### Library preparation and Illumina sequencing

Total DNA was quantitated using a Qubit HS DNA assay (Thermo Fisher). Sequencing libraries were constructed using 200 ng of input DNA. End repair, A-tailing and adapter ligation were performed using reagents from the SureSelect XT Reagent Kit (Agilent), each step followed by a cleanup procedure using AMPure XP beads (Beckman Coulter) at 1.8 x concentration. Following adapter ligation, four rounds of PCR (Herculase II, Agilent) were performed to enrich for correctly adapter-ligated fragments. Initial denaturation was at 98°C for 2 min, followed by 4 cycles of denaturation (98°C, 30 s), annealing (65°C, 30 s) and extension (72°C, 1 min), and finally a 10 min extension step at 72°C. Libraries were purified as described above and diluted 1:6 with nuclease free water. A second round of PCR (6 cycles) using 15 μl of input was performed using indexing primers available in the SureSelect XT Reagent Kit and PCR conditions as outlined above except for an adjustment to the annealing temperature (to 57°C). Final libraries were analysed using a Qubit Fluorimeter and Tapestation prior to sequencing across 8 runs on an Illumina MiSeq (2x150 bp PE reads, 300 cycle kit).

RNASeq libraries were prepared using the SureSelect Strand-Specific RNA Library Prep for Illumina Multiplexed Sequencing (Protocol version D0, 2015), multiplexed and sequenced on a single Illumina NextSeq run (2x34 bp PE reads, 75 cycle v2 high output).

### Sequence data analyses

Sequence run data were de-multiplexed using bcl2fastq2 v2.17 under stringent conditions (—barcode mismatches 0). Demultiplexed datasets were trimmed using TrimGalore software (http://www.bioinformatics.babraham.ac.uk/projects/trim_galore/) to remove low quality 3’ ends and aligned against the HSV-1 strain 17 genome (NC_001806) using BWA [57]. Genome-wide coverage and read depth data were calculated using custom scripts following the generation of pileup files using SAMTools [58]. RNASeq data were processed as above, except that alignment of reads against the HSV-1 strain 17 genome was performed using the splice-aware BBMap (http://sourceforge.net/projects/bbmap/) with default parameters.

#### DNA per fraction

Percentage of HSV-1 DNA to total DNA was calculated by the ratio of HSV-1 DNA reads to total DNA reads in each fraction. HSV-1 DNA genome copy equivalents in each fraction were then calculated by multiplying the genome copy equivalents in the total DNA by the percentage of HSV-1 DNA in each fraction. The number of HSV-1 genome copy equivalents in each fraction was divided by the number of genome copy equivalents in the total undigested and unfractionated HSV-1 DNA to give the percentage of HSV-1 genome copy equivalents recovered in each fraction, or to the addition of the number of HSV-1 genome copy equivalents in all fractions and pellet to give the percentage of HSV-1 genome copy equivalents in each fraction.

#### Normalized DNA reads

DNA reads (250 bp window) were generated for the entire HSV-1 genome for each fraction. Total DNA reads of soluble and insoluble chromatin at any given genomic position were divided to the DNA reads in the unfractionated chromatin at the same position, to give the normalized DNA reads for that position in each chromatin fraction.

#### HSV-1 DNA locus in each fraction

HSV-1 DNA reads in each fraction for each position were corrected by the number of HSV-1 genome copy equivalents in that fraction. The corrected HSV-1 DNA reads in each position were then normalized to the total HSV-1 DNA reads in that position in the same fraction, to give the number of HSV-1 genome copy equivalents in each genome position in every fraction. The sum of the number of HSV-1 genome copy equivalents in each position in all fractions was then normalized to the number of HSV-1 genome copy equivalents in the same position of the undigested and unfractionated chromatin, to give the recovery of each HSV-1 locus after digestion.

#### Gene sampling

The number of DNA reads equal or larger to one mapping to every 250 bp window was manually counted as 1. Then, the manually corrected reads were assigned to each gene according to their positions. Gene sampling was calculated as the sum of the manually corrected DNA reads of that gene, normalized to the sampling of the same gene in the undigested and unfractionated chromatin. Sampling of all genes in each kinetic class was averaged to give the average sampling of that kinetic class. Gene sampling of all genes in each fraction was averaged to give the mean sampling. Mean sampling in each fraction was divided by the overall mean sampling to analyze sampling enrichment in each fraction.

### CTCF chromatin immunoprecipitation

Vero cells (6 x 10^6^ on a 100 mm dish) were cross-linked with 5 ml of DMEM supplemented with 100 μl of 1M Na_2_HPO_4_ pH 8.96 and 142 μl of 37% formaldehyde, to a final concentration of 1%, for 10 min at 37°C. Crosslinking was quenched with 5 ml of DMEM supplemented with 714 μl of 2 M glycine, to a final concentration of 250 mM. After vacuuming, monolayers were further incubated with 5 ml of DMEM supplemented with 333 μl of 2 M glycine, to a final concentration of 125 mM, for 5 min at room temperature. Cells were then washed with 10 ml 4°C PBS supplemented with protease inhibitor (Pierce Protease Inhibitor Tablets EDTA-free [A32965]). Monolayers were scraped in 15 ml of 4°C PBS supplemented with protease inhibitor and transferred to a 50 ml conical tube; the dishes were then washed with other 15 ml of 4°C PBS, for a final volume of 30 ml. Cells were pelleted and resuspended in 6 ml of 4°C reticulocyte standard buffer (RSB, 10 mM Tris [pH 7.5], 10 mM NaCl, 5 mM MgCl_2_) supplemented with protease inhibitor. Cells were re-pelleted, resuspended in 6 ml 4°C RSB supplemented with protease inhibitor and incubated on ice for 20 min. The plasma membrane was lysed by adding 6 ml of RSB 1% triton X-100 supplemented with protease inhibitor and incubating on ice for 15 min, flipping the tubes upside down thrice. Nuclei were pelleted by centrifugation at 3,250 xg for 25 min at 4°C, resuspended in 1 ml of RSB and re-pelleted at 21,000 xg for 1 min at 4°C. Nuclei were resuspended and lysed in 1 ml chromatin extraction buffer (1 mM ethylene glycol-bis(beta-aminoethyl ether)-N, N, N’, N’,- tetra-acetic acid (EGTA), 2% Triton X-100, 3 mM MgCl2, 2 mM Tris [pH 8]) supplemented with protease inhibitor on ice for 60 min, pipetting up and down 10 times every 10 min. Chromatin was pelleted at 4,000 xg for 10 min at 4°C, resuspended in 1 ml of ChIP binding buffer (1% Triton, 10 mM Tris pH 8.0, 150 mM NaCl, 2 mM EDTA), sheared by sonication, flash frozen, and stored at -80°C.

Sheared chromatin was thawed on ice and diluted with ChIP binding buffer, to a final concentration of 16 ng/μl. Ten micrograms per ChIP reaction of CTCF antibody (07–729; EMD Millipore) or nonspecific IgG (12–370; EMD Millipore) were conjugated to 1.5 mg of Dyna Beads (Invitrogen 10001D) for ~20 hrs at 4°C, with constant rotation. Chromatin (8 μg) was incubated with bead-Ab complex for ~25 hrs at 4°C, with constant rotation. Immunopurified complexes were rinsed consecutively with 1 ml of each washing solution 1 (1% Triton, 2 mM EDTA, 20 mM Tris pH 8.0, 150 mM NaCl), 2 (2 mM EDTA, 20 mM Tris pH 8.0, 150 mM NaCl), 3 (1% Triton, 2 mM EDTA, 20 mM Tris pH 8.0, 500 mM NaCl), 4 (2 mM EDTA, 20 mM Tris pH 8.0, 500 mM NaCl), 5 (1% NP-40, 1% NaDOC, 1 mM EDTA, 10 mM Tris pH 8.0, 250 mM LiCl), and 6 (1 mM EDTA, 10 mM Tris pH 8.0, 250 mM LiCl). Immunopurified complexes were eluted with 600 μl of ChIP elution buffer (10 mM Tris-HCl pH 8.0, 150 mM NaCl, 1 mM EDTA, 0.5% SDS) and 6 μl of proteinase K (20 mg/ml), and then incubated at 65°C for approximately 19 hrs for decrosslinking and deproteinization. Immunoprecipitated or input (0.8 μg of chromatin) DNA was phenol-chloroform extracted, isopropanol precipitated, ethanol washed, and subjected to qPCR (Fast SYBR Green master mix) with 300 nM each the B9# (5’- TCCTGTTGATCGATACGGGG -3’, 5’- ATCACAAACACTGGCAGCAG -3’) B1# (5’- TCTAACGTTACACCCGAGGC -3’, 5’- GTATATATGCGCGGCTCCTG-3’), ICP4 (5’- GAGGATCCCCGCGGCGCCGTA -3’, 5’- TGGCCATGAGCCGCCGATACGAC -3’), CTRS3 (5’- CACGAACGACGGGAGCG -3’, 5’- CACCCAAGGTGCTTACC -3’), CTRS1/2 (5’- GCCCCTCGAATAAACAACGCTA -3’, 5’- GTTGTGGACTGGGAAGGCGC -3’), or CTa’m (5’- GGCTGCCACAGGTAAAACA -3’, 5’- TGTAGCAGGAGCGGTGTG -3’). DNA from the untreated infections was diluted 1:1,000. qPCR conditions (except for the ICP4 peak) were 20 sec denaturation at 95°C, followed by 40 cycles of 3 sec denaturation at 95°C and 30 sec annealing/elongation at 60°C, followed by 15 sec denaturation at 95°C and 60 sec annealing/elongation at 60°C, followed by a temperature ramp from 60 to 95°C in 1% increments every 15 sec and a final 15 sec incubation at 60°C. The ICP4 peak was amplified by 20 sec denaturation at 95°C, followed by 40 cycles of 3 sec denaturation at 95°C and 30 sec annealing/elongation at 70°C, followed by 15 sec denaturation at 95°C and 60 sec annealing/elongation at 60°C, followed by a temperature ramp from 60 to 95 in 1% increments every 15 sec and a final 15 sec incubation at 60°C. Viral copy number was calculated from a standard curve using 10 fold dilutions of 300,000 genome copies of cell free HSV-1 DNA in 2.8 ng of purified Vero cell DNA and sheared by sonication. Immunoprecipitation efficiencies were calculated by subtracting the genome copy equivalents precipitated with the non-specific IgG from those precipitated with α-CTCF antibody and then dividing by the total genome copy equivalent in the input.

## Results

### Most intracellular HSV-1 DNA is neither protein-free nor encapsidated at 7 hours after infection

**Fig 1:**
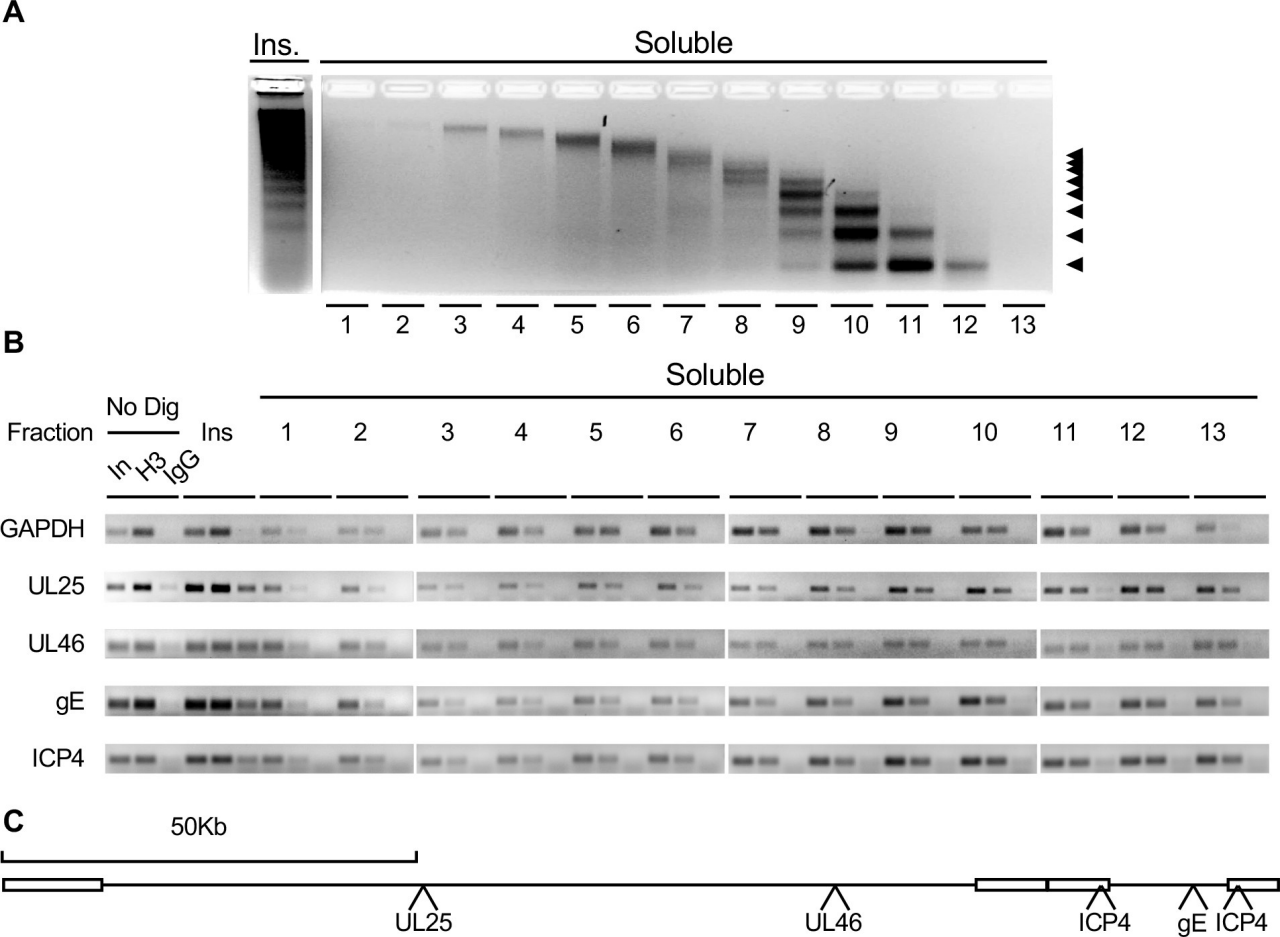
At after infection, HSV-1 DNA was protected from serial MCN digestion to sizes of mono to poly-nucleosomes that fractionated in complexes with the hydrodynamic ratios of mono to poly-nucleosomes and contain histone H3. Nuclei of infected cells were subjected to serial MCN digestion and then cross-linked. (A) Cross-linked soluble chromatin separated by hydrodynamic ratios in a 0–10% sucrose gradient, and resolved in 2% agarose gel electrophoresis, stained with EtBr. The left pointing arrowheads at the right indicate the migration of mono- to octa-nucleosome-sized DNA. (B) Chromatin immunoprecipitation of HSV-1 DNA with histone H3. EtBr stained agarose gels of saturating PCR-amplified cellular (GAPDH) or HSV-1 (UL25, UL46, ICP4, or gE) DNA co-immunoprecipitated with histone H3. (C) Cartoon presenting the positions of the PCR amplicons in the HSV-1 genome. In, input (2%); H3, anti-histone H3 antibody; IgG, isotype antibody control (labeled only in the No Dig sample for simplicity); No Dig, undigested unfractionated chromatin; Ins, insoluble chromatin.

Prior studies had suggested that HSV-1 DNA is organized in highly dynamic nucleoprotein complexes that otherwise behave like cellular chromatin, as evidenced by hybridization of chromatin resolved after serial micrococcal nuclease digestion [5, 6]. However, it is also possible that protein-free HSV-1 DNA simply co-fractionated with cellular chromatin. To test for this possibility, the DNA in each fraction was co-immunoprecipitated with histone H3. The fractionation pattern of the soluble chromatin was not obviously affected by cross-linking (Fig 1A). At 7 hours after infection, HSV-1 DNA from the immediate-early (ICP4), early (gE) or late (UL25 and UL46) genes co-immunoprecipitated with histone H3 in all fractions, as tested by saturating PCR (Fig 1B). The co-immunoprecipitation of both cellular and viral DNA in the most inaccessible fractions was less efficient than in the more accessible ones. Intracellular HSV-1 DNA thus fractionated in complexes with the hydrodynamic ratios of mono- to poly-nucleosomes in which it interacts with histone H3.

**Fig 2:**
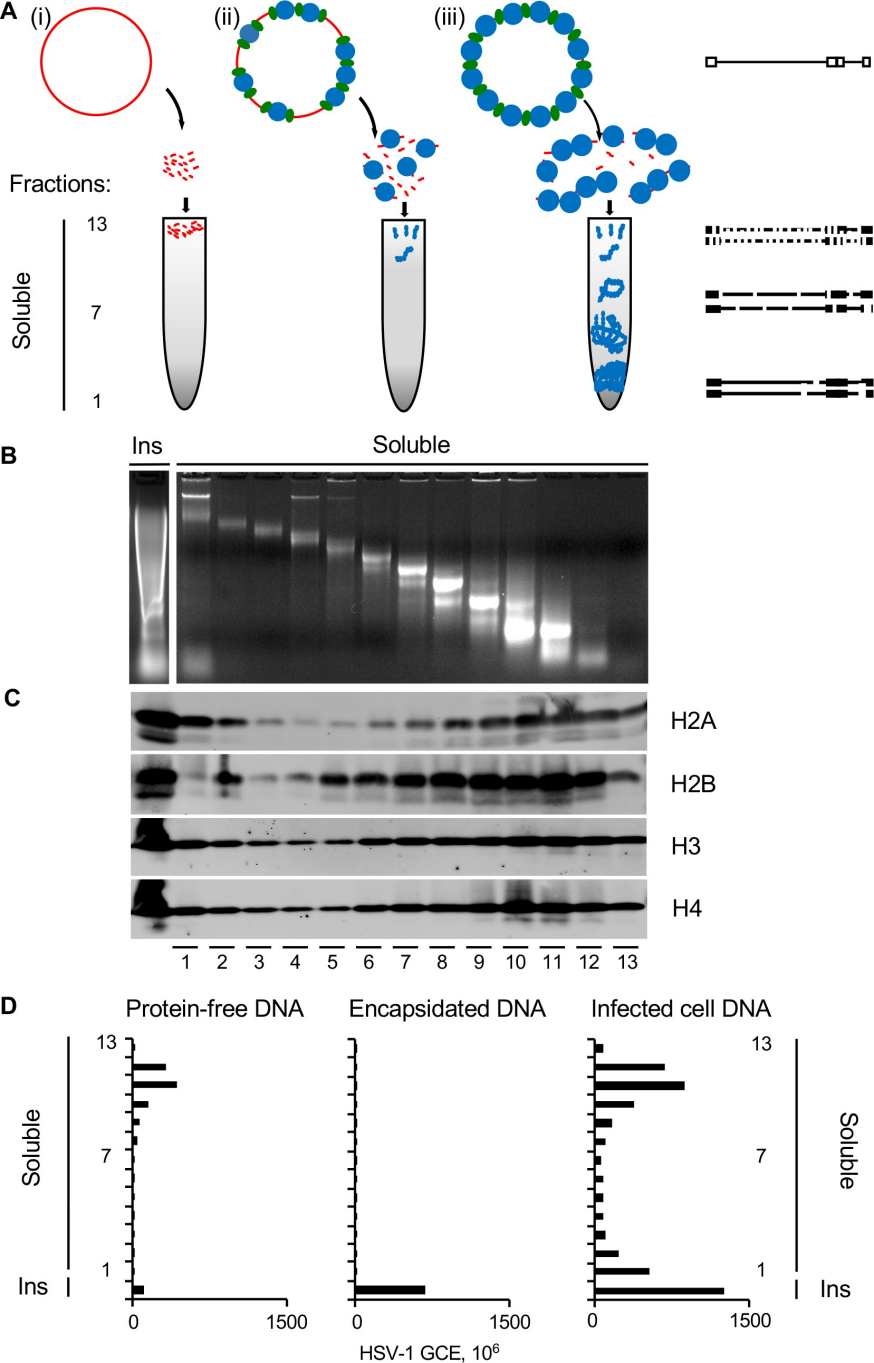
Intracellular HSV-1 DNA is differentially protected from MCN digestion than deproteinized or encapsidated DNA. (A) Cartoon presenting the different models proposed for the HSV-1 chromatin and the expected fractionation patterns after serial digestion and sucrose gradients of: (i) mostly non-chromatinized HSV-1 DNA, (ii) HSV-1 DNA associated with nucleosomes only sporadically, (iii) fully (stably or unstably) chromatinized HSV-1 DNA. (B) DNA from each fraction was resolved in 2% agarose gel electrophoresis and stained with EtBr. (C) Western blot for core histones (H2A, H2B, H3 and H4) in all fractions, each resolved by SDS-PAGE and probed with corresponding antibodies. (D) Bar graph showing the number of HSV-1 genome copy equivalents in each fraction. Chromatin of HSV-1 infected cells was digested and fractionated. DNA in each fraction was subjected to deep sequencing. Ins, insoluble chromatin; GCE, genome copy equivalents. Results from one experiment representative of three.

Knowing that the HSV-1 DNA in all fractions was protected to nucleosome sizes in complexes with the hydrodynamic ratios (and therefore shape and size) of cellular mono- to poly-nucleosomes and which contain histone H3, we next evaluated the distribution of protein-free, encapsidated and intracellular HSV-1 DNA after serial MCN digestion by differential centrifugation and deep sequencing (Fig 2A). Any protein-free HSV-1 DNA would be digested to small fragments, which would fractionate mostly to the very top of the gradients, or to short polynucleotides, which would not be detected by sequencing (Fig 2A). Sporadic nucleosomes would partially protect HSV-1 DNA and result in digestion to mono- to short poly-nucleosomes which would fractionate to only the top fractions of the gradient (Fig 2A). Fully (stably or unstably) chromatinized HSV-1 DNA would be sufficiently protected to limited digestion to result in mono- to long poly-nucleosomes, which would fractionate throughout the gradients (Fig 2A). Cellular chromatin resolved as mono- to poly-nucleosomes throughout the entire gradient, as expected, with DNA and all core histones present in each fraction (Fig 2B and 2C).

DNA in each chromatin fraction and in the unfractionated chromatin was then subjected to deep sequencing. The fraction of viral to total (cellular plus viral) reads and the total HSV-1 genome copy equivalent were used to calculate the number of HSV-1 genome copy equivalents in each fraction (Fig 2D). The fractionation of protein-free and encapsidated DNA marked the migration of naked or fully protected DNA, respectively. Protein-free HSV-1 DNA (approximately 800 genome copy equivalents per cell) added to chromatin of mock infected cells was poorly protected and resolved almost exclusively to the lowest density fractions (10 to 12—Fig 2D). Encapsidated HSV-1 DNA (approximately 800 genome copy equivalents per cell) added to the chromatin of mock infected cells resolved to the highest density fractions (Fig 2D), clearly peaking with the insoluble chromatin as expected.

There were approximately 845 HSV-1 genome copy equivalents per cell at 7 hours after infection, constituting 6% of the nuclear DNA. Sixty percent of all the intranuclear HSV-1 DNA was recovered after MCN digestion and fractionation, which is consistent with previous results using hybridization [5, 6]. In contrast to protein free or encapsidated HSV-1 DNA, intracellular HSV-1 DNA migrated throughout the gradient. Nonetheless, it peaked in fractions 9 to 13, with the major peak in fractions 10 to 12, containing the smallest nucleosome complexes (i.e. the most accessible chromatin) and fraction 1 and the insoluble chromatin, containing the largest poly-nucleosome complexes (i.e. the least accessible chromatin) (Fig 2D).

**Fig 3:**
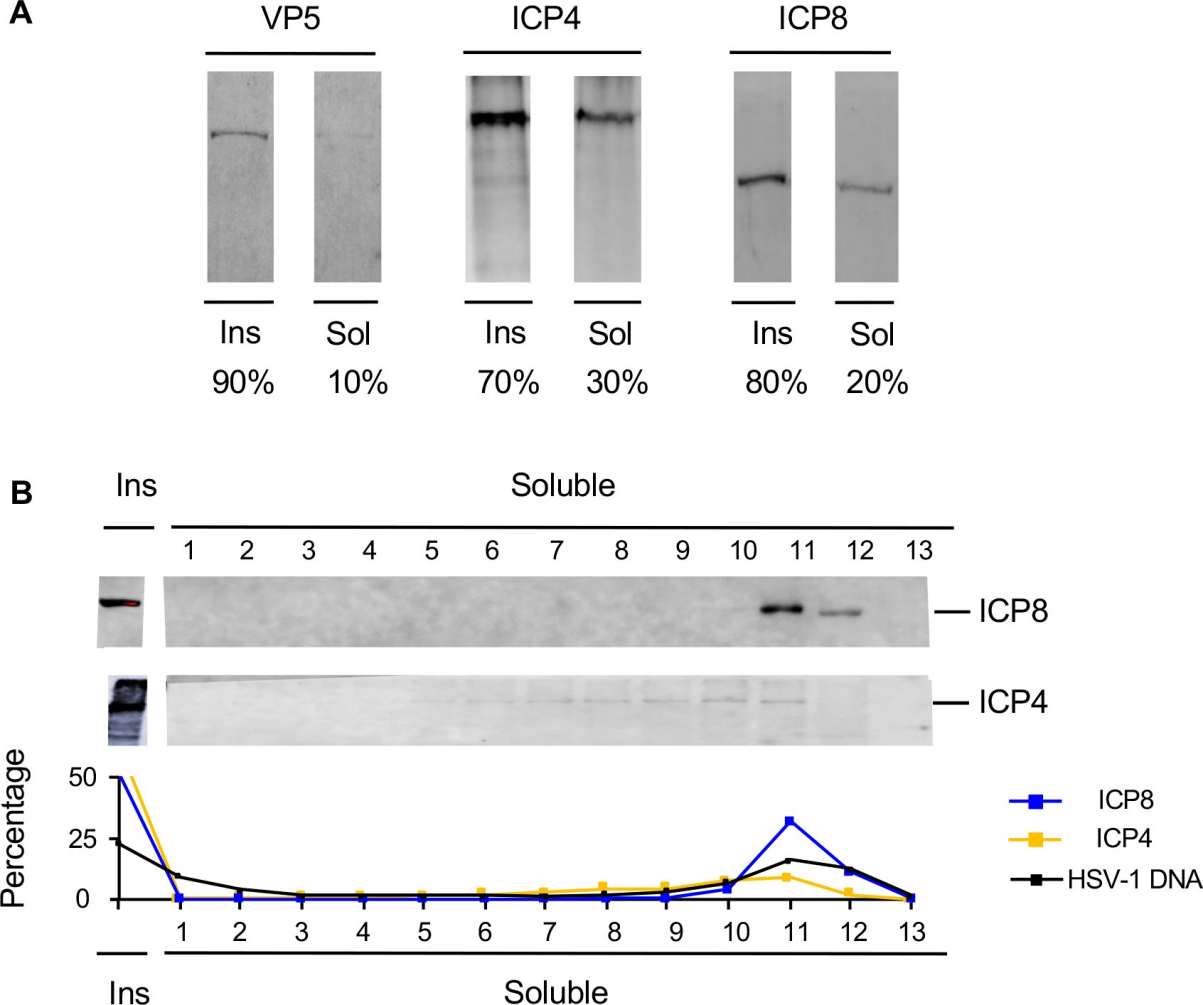
Fractionation of the major HSV-1 DNA binding proteins. (A) Western blots of VP5, ICP4 and ICP8 in the insoluble and soluble chromatin fractions. Results representative of two independent repeats. (B) Fractionation of ICP4, ICP8, and HSV-1 DNA after serial MCN digestion and sucrose gradient centrifugation. Western blots of ICP4 and ICP8, and line graph presenting the percentage of ICP4, ICP8 and HSV-1 DNA in each fraction. VP5 was below sensitivity levels in all fractions. Ins, insoluble chromatin fraction; sol, soluble chromatin fraction. Average of three (ICP8) or two (ICP4) independent experiments.

We considered the possibility that a large fraction of the protected HSV-1 DNA was still encapsidated. However, VP5 was hardly detectable (~10%) when all soluble chromatin was analyzed together (Fig 3), and undetectable in any individual fraction (S7 Fig). ICP4 and ICP8 are two major HSV-1 DNA binding proteins. Approximately 30% ICP4, and 20% ICP8 resolved in the soluble chromatin, while approximately 70% and 80%, respectively, was present in the insoluble chromatin. By contrast, only ~25% HSV-1 DNA was in the insoluble chromatin at this time. The HSV-1 DNA present in the insoluble chromatin may thus have been protected in part by ICP4 or ICP8 (or other non-chromatin proteins). Most ICP8 in the soluble chromatin resolved to fractions 10 to 12 (Fig 3), whereas ICP4 more evenly fractionated through fraction 5 to 13.

### Transcribed and non-transcribed HSV-1 genes are in similarly accessible chromatin

Besides other effects such as recruitment of transcription factors, differential chromatinization of individual HSV-1 genes could regulate the accessibility of each gene and therefore its transcription. To modulate the transcription level of specific groups of genes, we used cycloheximide (CHX) and roscovitine (Rosco). Cycloheximide inhibits protein synthesis, thereby indirectly inhibiting transcription of most HSV-1 genes while allowing high levels of transcription of the IE ones. Rosco inhibits transcription of all HSV-1 genes. When E and L gene transcription was inhibited with CHX, there were approximately 35 HSV-1 genome copy equivalents per cell (infected at an moi of 10) at 7 hpi. When HSV-1 transcription was inhibited with Rosco, there were approximately 42 HSV-1 genome copy equivalents per cell. As discussed, there were approximately 845 HSV-1 genomes per cell at 7 hpi in the untreated infections.

**Fig 4:**
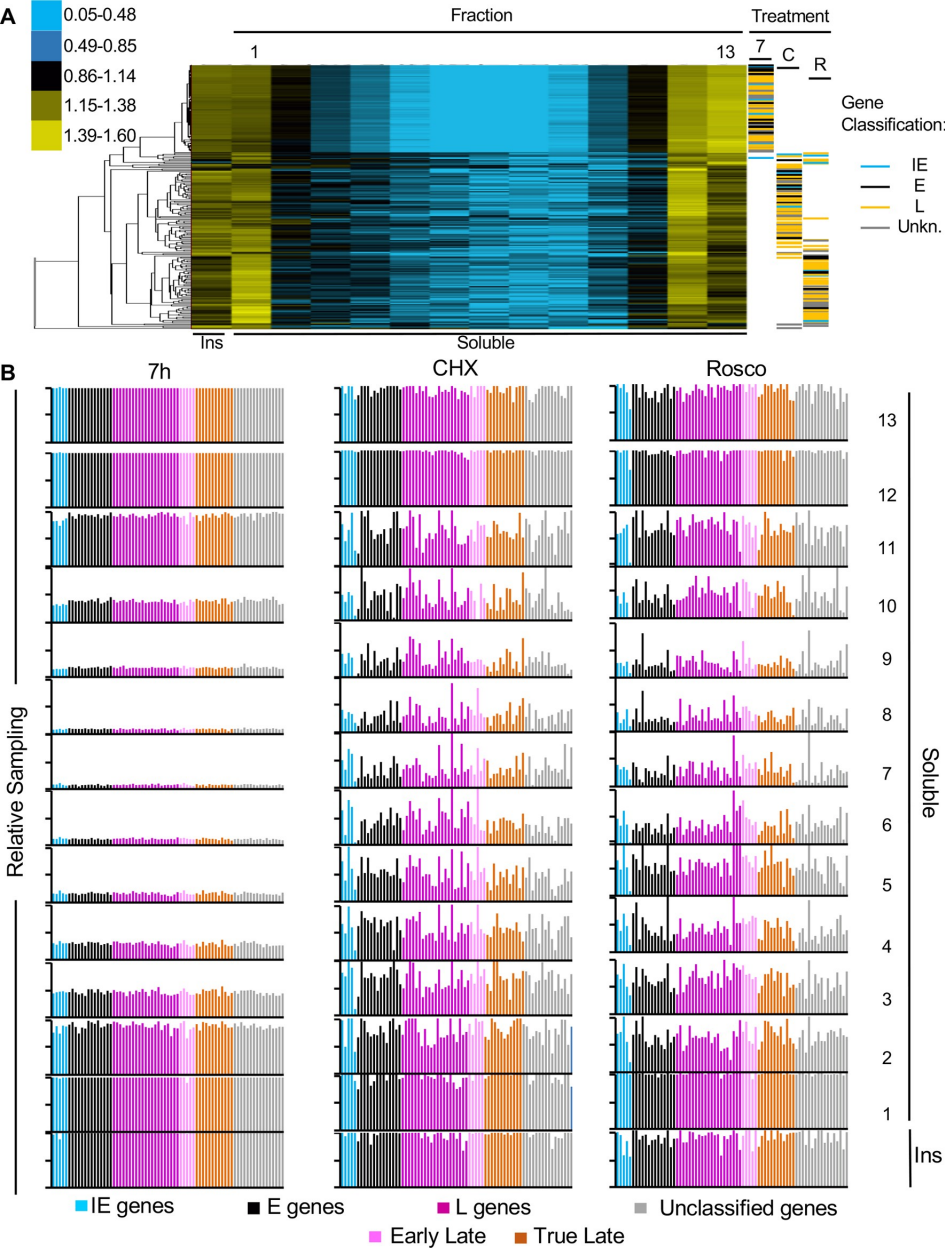
All HSV-1 genes resolved together to the least and most accessible chromatin fractions. (A) Cluster analyses of the number of DNA reads for IE, E or L genes in each fraction. Cluster analysis was performed with Cluster 3.0 from Stanford University, visualized with Java Treeview. Color key on the left indicates ratio of reads in fraction over reads in the total DNA (ranges are in log_2_). 7, 7 hpi, untreated infections; C, CHX- treated infections (7 hpi); R, roscovitine-treated infections (7 hpi). (B) Gene sampling of each HSV-1 gene in each fraction. Sampling of each HSV-1 gene was calculated and normalized to the sampling of the same gene in the undigested and unfractionated chromatin. Each gene is color coded according to kinetic class. Blue, IE genes; black, E genes; dark purple, unclassified L genes; light purple, early L genes; brown, true L genes; grey, unclassified genes. Ins, insoluble chromatin fraction. Results from one experiment representative of three.

To evaluate the relative accessibility of the IE, E or L genes, the number of DNA reads for each IE, E or L gene in each fraction were subjected to cluster analysis (Fig 4A). If transcription of a specific gene was related to its accessibility, then the transcribed IE genes in infections treated with CHX would cluster together with all transcribed genes in untreated infections, whereas the non-transcribed E and L genes in CHX-treated infections would cluster with all non-transcribed genes in the infections treated with Rosco. However, all IE, E and L genes in the untreated infections were equally overrepresented in the least and most accessible chromatins (with the caveat that the top fractions may also contain some naked DNA that was somewhat not digested by MCN) and thus clustered together, and away from IE, E and L genes in the infections treated with Rosco or CHX. The IE, E and L genes in the infections treated with CHX also clustered together, as they were more evenly distributed in all fractions than in the untreated infections (Fig 4A and 4B). They were less depleted in the intermediate fractions and less overrepresented in the most accessible fractions. The DNA of some IE and E genes had a somewhat unique distribution among all genes in the infections treated with Rosco, clustering closer to genes in the CHX-treated infections. Therefore, the accessibility of a given gene, at the population level, does not directly correlate with its transcription levels.

To further test whether the accessibility of any individual gene was related to its transcription, we evaluated individually the fractionation of each known HSV-1 gene. For the accessibility of a gene to be related to its transcription level, the entire gene (but not necessarily its promoter) would be expected to be similarly accessible. Consequently, the entire gene would fractionate as a unit in the relevant fractions and deep sequencing would sample DNA sequences covering most of the gene in those fractions. As HSV-1 genes range from 170bp to 9,490bp, we analyzed the relative sampling of each gene in each chromatin fraction or in the unfractionated chromatin. The fraction of a given gene sampled in each fraction was then normalized to the fraction of the same gene sampled in the unfractionated chromatin and expressed as percentage (gene sampling).

The sampling of each gene was calculated in each fraction and plotted according to their kinetic groups (Fig 4B). At 7 h after infection, the least (insoluble chromatin and fractions 1 and 2) and most (fractions 11 to 13) accessible chromatin were enriched in fully sampled HSV-1 genes, whereas the intermediately accessible chromatin contained only random samplings of each gene. Fractions 3 and 10 sampled approximately half of each HSV-1 gene, whereas the rest of the intermediate fractions had sampled only less than 20% of each gene. No individual HSV-1 gene was differentially sampled in any fraction.

When transcription of E and L genes was inhibited (in infections treated with CHX), the transcriptionally active IE genes still fractionated as the transcriptionally inactive E and L genes (Fig 4B). The least (insoluble chromatin and fraction 1) and most (fractions 12 to 13) accessible chromatin were enriched in completely sampled HSV-1 genes. In the intermediately accessible chromatin each gene was sampled less frequently—albeit not nearly to the same extent as in the untreated infections. Fractions 2 to 5 and fraction 11 sampled 40–60% of each HSV-1 gene, and fractions 6 to 10, 25% - 40%.

No HSV-1 genes fractionated differently when HSV-1 transcription was inhibited with Rosco either, and the least (insoluble chromatin and fraction 1) and most (fractions 12 to 13) accessible chromatin were enriched in mostly fully sampled HSV-1 genes (Fig 4B). As in the infections treated with CHX, the depletion in fully sampled genes in the intermediately accessible chromatin was only partial. Fractions 2 to 4 and fraction 11 sampled 40–60% of each HSV-1 gene, and factions 5 to 10, 25–40%. The depletion in fully sampled genes in the intermediate accessible chromatin could have been an artifact resulting from too little HSV-1 DNA. However, fully sampled HSV-1 genes resolved to the insoluble chromatin, and to fractions 1, 12, 13 and the insoluble chromatin in infections treated with CHX or Rosco. These fractions contained as little HSV-1 DNA as the intermediate fractions in the untreated infection, which contained only partially sampled genes. From the comparison with the CHX-treated infections, the accessibility of all genes at the population level related to the overall levels of transcription of the genomes, whereas the accessibility of any single gene, at the population level, did not.

**Fig 5:**
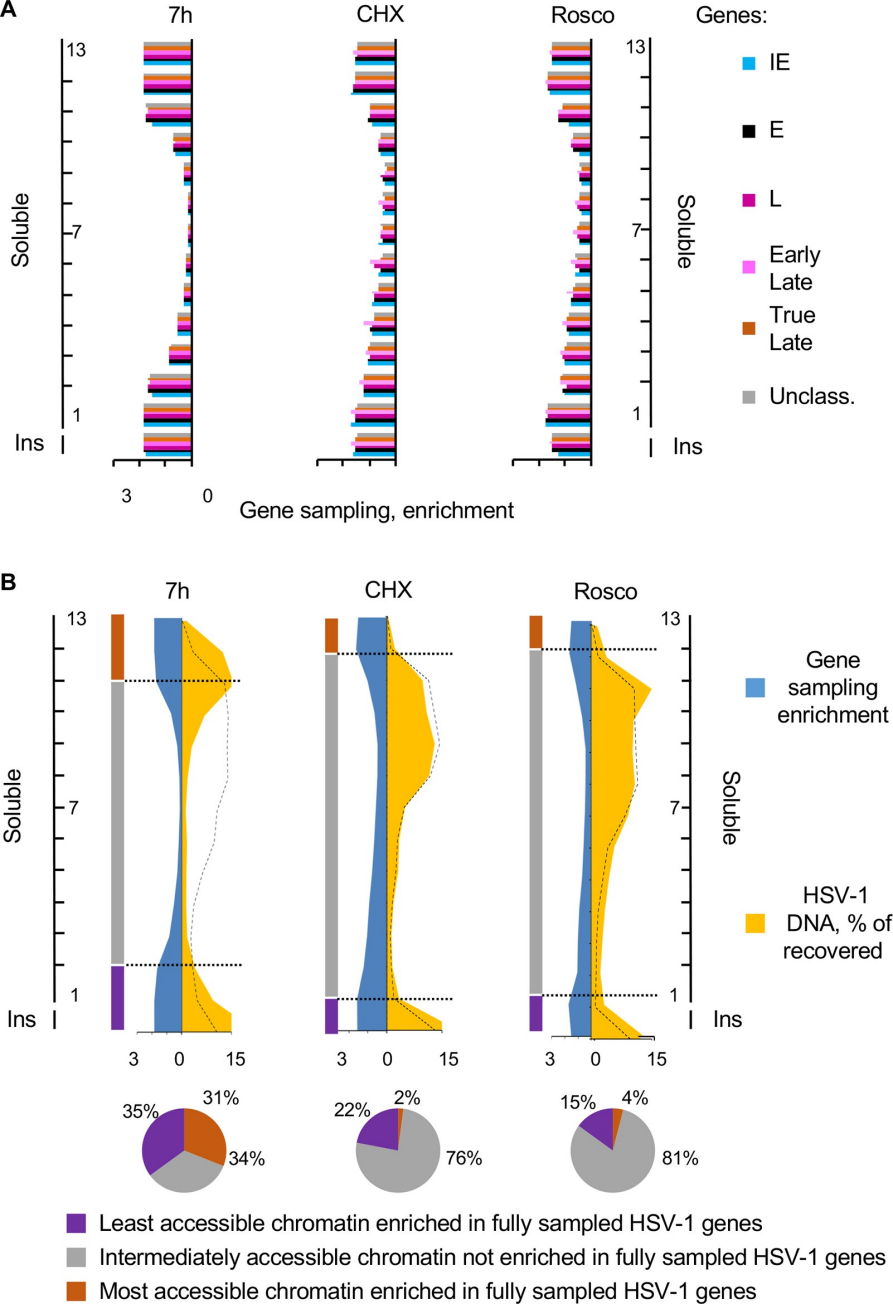
The most and least accessible chromatin enriched in fully sampled HSV-1 genes contained more HSV-1 DNA when transcription was active. (A) Bar graphs showing average sampling of all genes in each HSV-1 gene kinetic groups in each fraction. Sampling of each HSV-1 gene was calculated and normalized to the sampling of the same gene in the undigested and unfractionated chromatin. The normalized sampling of all genes in each group was then averaged. Blue, IE genes; black, E genes; dark purple, unclassified L genes; light purple, early L genes; brown, true L genes; grey, unclassified genes. (B) Area graphs and pie charts presenting relative enrichment in gene sampling and percentage of HSV-1 DNA in each fraction. Gene sampling in each fraction is expressed as ratio to the average sampling to present the relative enrichment in fully sampled genes in each fraction. The distribution of the cellular chromatin is indicated by the dotted black line. Pie charts indicating the percentages of the HSV-1 DNA in the most accessible chromatin fractions containing completely sampled HSV-1 genes (brown), in the intermediate accessible chromatin fractions containing random samplings of each gene (grey), or in the least accessible chromatin fractions containing completely sampled HSV-1 genes (purple). Ins, insoluble chromatin fraction. Results from one experiment representative of three.

To evaluate the global accessibility of IE, E and L genes, the average gene sampling for each group was calculated in each fraction. At 7 h after infection, the least and most accessible chromatins were enriched in fully sampled HSV-1 genes of all three kinetic classes. The other fractions were in contrast partially depleted of fully sampled genes (Fig 5A). The enrichment in fully sampled IE, E, or L genes in only the least and most accessible chromatins was not altered when only IE genes were transcribed to high levels (CHX), no gene was transcribed to high levels (Rosco), or all IE, E and L genes were transcribed to high levels (Fig 5A). In contrast, the depletion in fully sampled genes in the intermediately accessible chromatin was less marked in CHX or Rosco-treated infections. We concluded that transcription and accessibility of genes in any kinetic class are not directly related (Figs 4 and 5).

Sampling of all genes in all fractions was averaged. Sampling of all genes in each fraction was then expressed as enrichment over the average sampling and plotted together with the percentage of HSV-1 DNA in each fraction (Fig 5B). At 7 h after infection, the least (insoluble chromatin to fraction 2) and most (fractions 11 to 13) accessible chromatins that were enriched in fully sampled HSV-1 genes had most of the HSV-1 DNA. The most accessible chromatin that was enriched in fully sampled genes had 31% of the HSV-1 DNA, and the least accessible chromatin that was equally enriched in fully sampled genes, 35%. Although the fractions containing the most accessible chromatin could also contain some naked DNA, the presence of fully sampled genes indicates that the DNA in these fractions had not been digested extensively, contrary to what would had been expected from naked DNA. The intermediately accessible chromatin (fractions 3 to 10), which contained 34% HSV-1 DNA, contained only partially sampled genes (Fig 5B). This depletion in fully sampled genes was not a result of a mix of chromatinized and protein-free HSV-1 DNA. Protein-free HSV-1 DNA did not resolve to these intermediate fractions (Fig 2D) and DNA from an E and a L gene in these fractions co-immunoprecipitated with histone H3 (Fig 1B). The distribution of HSV-1 DNA was markedly distinct from that of the cellular DNA (dashed black line), indicating biophysical differences between the viral and cellular chromatin under these conditions.

When most HSV-1 transcription was inhibited with CHX, the least accessible chromatin that was enriched in fully sampled HSV-1 genes (insoluble chromatin and fraction 1) had 22% of the HSV-1 DNA. In contrast, the most accessible chromatin that was enriched in fully sampled genes (fractions 12 and 13) had only 2% of the HSV-1 DNA (Fig 5B). Similarly, when HSV-1 transcription was inhibited with Rosco, the least accessible chromatin that was enriched in fully sampled HSV-1 genes (insoluble chromatin and fraction 1) had 15% of the HSV-1 DNA whereas the most accessible chromatin that was also enriched in fully sampled genes (fractions 12 and 13), had only 4% of the HSV-1 DNA (Fig 5B). The intermediately accessible chromatin, containing only partially sampled genes, contained 75–80% of the HSV-1 DNA. In both conditions, the depletion in fully sampled genes in the intermediately accessible chromatin was only partial and progressive, with less depletion in the least accessible intermediate chromatin (fractions 1–6) and more in the most accessible ones (fractions 7–9). In both conditions, the distribution of the viral DNA very much replicated that of the cellular DNA, indicating similar biophysical properties between the viral and cellular chromatin when most of the viral genome is not transcribed.

In summary, the HSV-1 chromatin had similar biophysical properties to the cellular chromatin when there was limited HSV-1 transcription, but it was far more accessible when the genomes were transcribed. Moreover, the levels of HSV-1 DNA in the most accessible chromatin that was enriched in fully sampled genes related to the overall transcription level of the genomes.

### Changes in the accessibility of HSV-1 chromatin along infection

**Fig 6:**
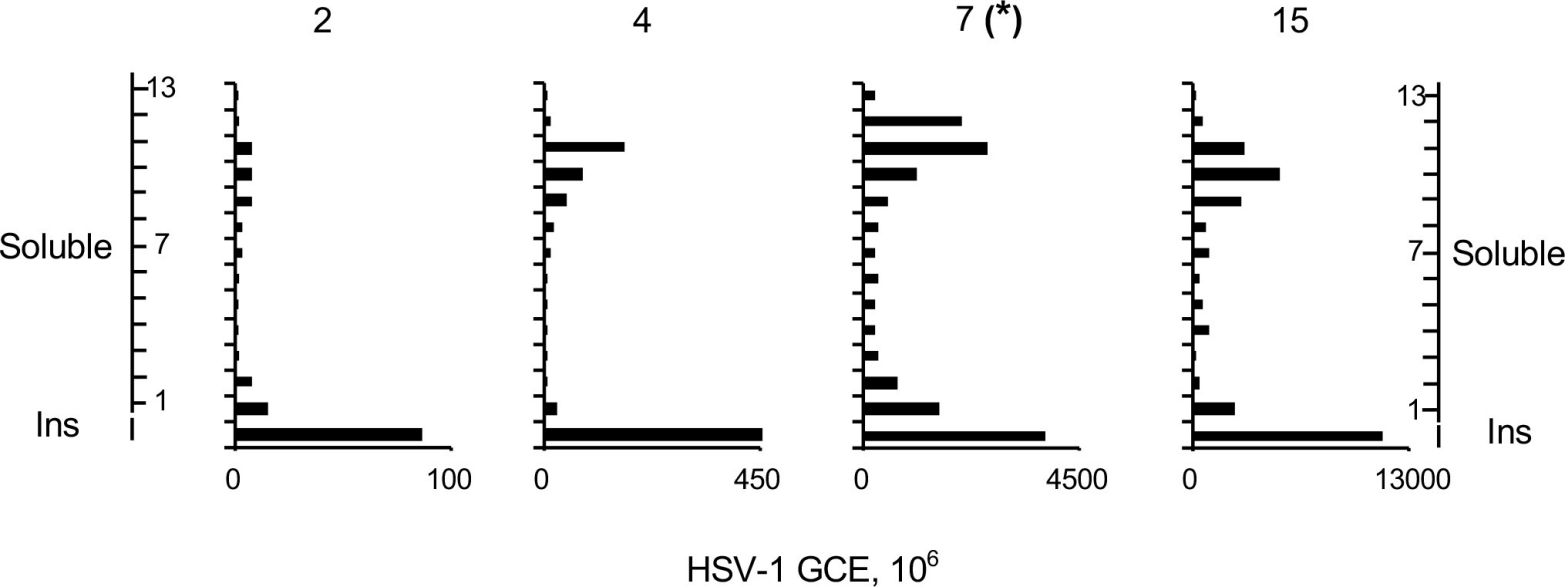
Differential fractionation of HSV-1 DNA throughout infection. Bar graphs showing HSV-1 genome copy equivalents in each fraction. (*) Data from Fig 2 presented again for comparison. Ins, insoluble chromatin fraction; GCE, genome copy equivalent. Results from one experiment representative of two.

We next evaluated the HSV-1 chromatin as infection progressed. At 2h after infection with 10 pfu/cell, there were approximately 20 HSV-1 genome copy equivalents per cell, constituting 0.2% of the DNA in the cell. The HSV-1 DNA was enriched in the least accessible chromatin (insoluble chromatin and fractions 1–2), with a very minor relative enrichment in the most accessible chromatin (fractions 9–11), and depleted in the intermediately accessible chromatin (fractions 3–8) (Fig 6). The distribution of the HSV-1 DNA changed at 4h, when there were approximately 90 HSV-1 genome copy equivalents per cell, constituting 0.8% of the nuclear DNA. HSV-1 genomes were then enriched in the least (mostly in the insoluble chromatin) and in the most (fractions 9–11) accessible chromatin, while they were relatively depleted in the intermediately accessible chromatin (fractions 2–8). As already discussed, there were approximately 850 HSV-1 genome copy equivalents per cell, constituting 6% of the nuclear DNA at after infection. The HSV-1 genomes were also enriched in the most and, less so, in the least accessible chromatin and depleted in the intermediately accessible chromatin (Fig 2D–presented again in Fig 6 for comparison). At 15h after infection, there were approximately 2,650 HSV-1 genome copy equivalents per cell, constituting 20% of the nuclear DNA. At this time, the HSV-1 DNA was also enriched in the least (insoluble chromatin and fraction 1) and most (fractions 9–11) accessible chromatin (Fig 6).

### The accessibility of all HSV-1 genes increases in synchrony as the infection progresses

No particular HSV-1 gene was differentially accessible at 7 hpi, regardless of whether transcription was inhibited or not (Fig 4), and the most accessible chromatin was enriched in fully sampled genes only when there was abundant transcription (Fig 5B). Both the most and least accessible chromatins contained fully sampled HSV-1 genes (Fig 4). One possibility was that the most accessible chromatin contained transcriptionally competent genomes and the least accessible one, transcriptionally silenced ones (and potentially encapsidated DNA although VP5 was barely detectable at this time), whereas the intermediately accessible chromatin contained genomes transitioning between states. Then, the fractionation of the HSV-1 genomes would be expected to change from the least accessible chromatin toward the most accessible one as infection progresses. To test this model, we analyzed the accessibility of the viral chromatin as the infection progressed.

**Fig 7:**
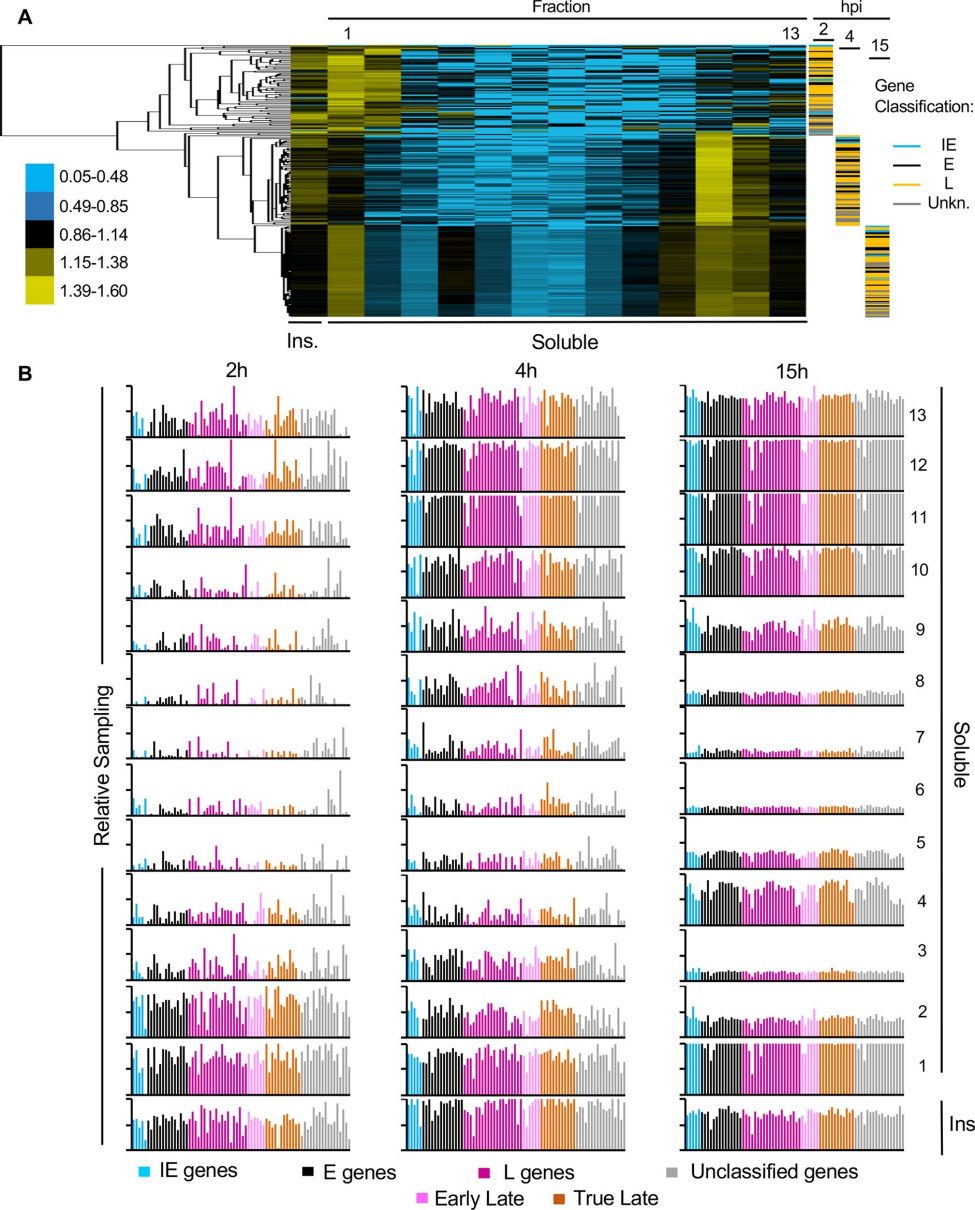
All HSV-1 genes resolved together to the least and most accessible chromatin fractions as infection progressed. (A) Cluster analyses of the number of DNA reads from IE, E and L genes in each fraction. Cluster analysis was performed using Cluster 3.0 from Stanford University, visualized with Java Treeview. (B) Bar graphs showing sampling of each HSV-1 genes at 2h, 4h, and 15h after infections. Sampling of each HSV-1 gene was calculated and normalized to the sampling of the gene in the undigested and unfractionated chromatin. The sampling of each gene was plotted according to kinetic class. Blue, IE genes; black, E genes; dark purple, unclassified L genes; light purple, early L genes; brown, true L genes; grey, unclassified genes. Ins, insoluble chromatin fraction. Results from one experiment representative of two.

When evaluated by their fractionation, IE, E and L genes clustered together according to time after infection, not to kinetic class (Fig 7A). All IE, E, and L genes were relatively underrepresented in the most accessible chromatin and thus clustered the furthest away at 2 h, when there is limited HSV-1 transcription. IE, E and L genes were all enriched in the most and least accessible chromatin at 4 h, when there is already widespread HSV-1 transcription. At 12 h, the depletion in the intermediately accessible chromatin was even more uniform and marked. Genes of all kinetic classes resolved similarly and were similarly sampled in each fraction at all infection times (Fig 7B).

**Fig 8:**
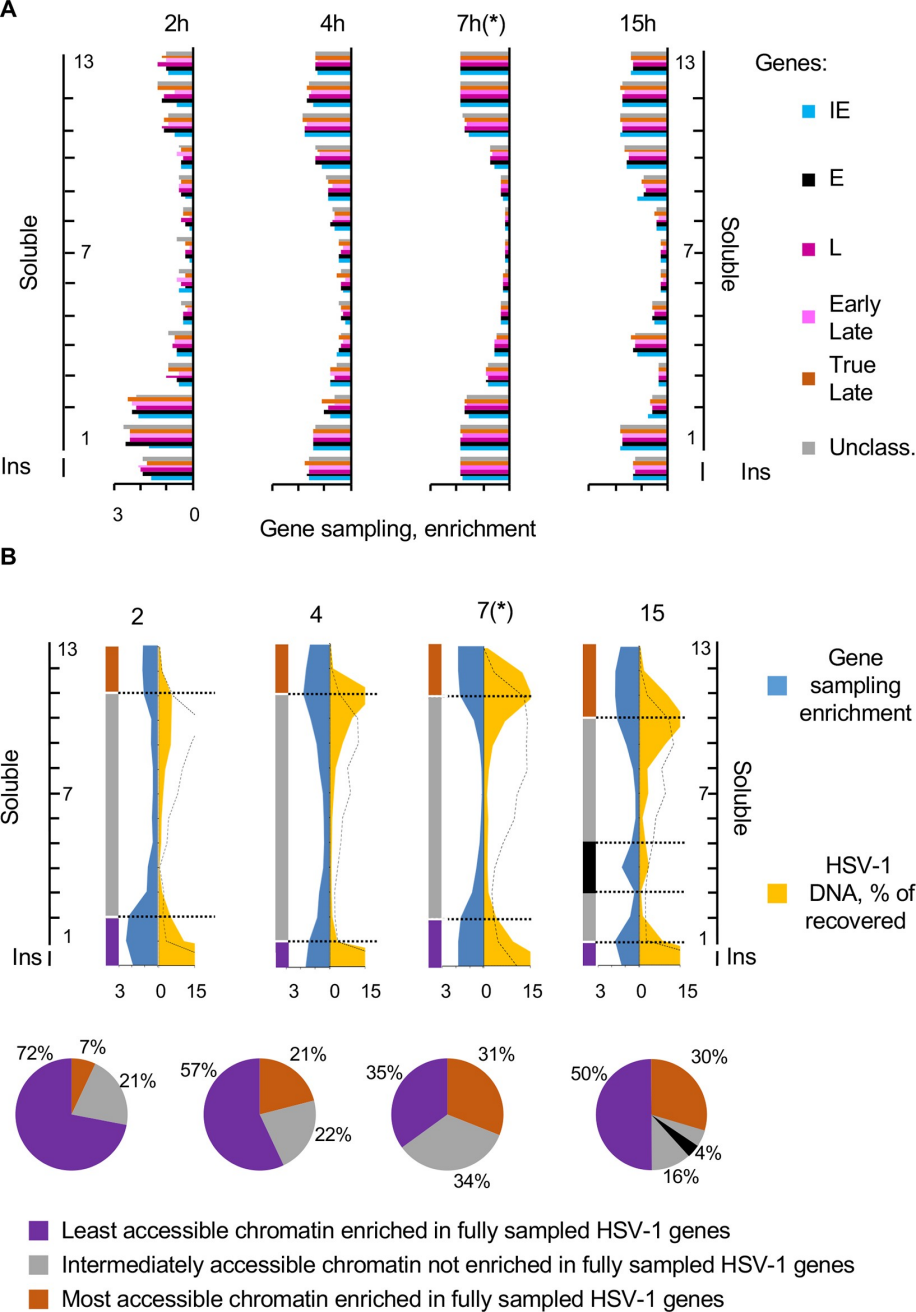
The most and least accessible chromatin enriched in completely sampled HSV-1 genes contained more HSV-1 DNA as the infection progressed. (A) Bar graphs showing average sampling of the genes in each HSV-1 gene kinetic class in each fraction at 2, 4 and 15 hours after infection. Sampling of each HSV-1 gene was calculated and normalized to the sampling of the gene in the undigested and unfractionated chromatin. The normalized sampling of all genes in each group was then averaged. Blue, IE genes; black, E genes; dark purple, unclassified L genes; light purple, early L genes; brown, true L genes; grey, unclassified genes. (B) Area graphs showing relative enrichment in completely sampled genes and percentage of HSV-1 DNA in each fraction. Gene sampling in each fraction is expressed as ratio to the average sampling (relative enrichment). The distribution of the cellular chromatin is indicated by the dotted black line. (*) Data from Fig 5 presented again for comparison. Pie charts showing percentage of HSV-1 DNA in the most accessible chromatin fractions containing completely sampled HSV-1 genes (brown), in the intermediate accessible chromatin fractions containing random sampling of each gene (grey), or in the least accessible chromatin fractions containing completely sampled HSV-1 genes (purple). Ins, insoluble chromatin fraction. Results from one experiment representative of two.

To analyze potential differences among IE, E or L genes, we averaged the sampling of all genes in each kinetic class. At 2 h, the least accessible chromatin fractions (insoluble, fractions 1 and 2) were enriched in fully sampled HSV-1 genes of all kinetic classes (Fig 8A). The fully sampled genes were depleted in the intermediately accessible fractions, although not to the extent as in 7 h infections. The most accessible chromatin fractions (fractions 11 to 13) were only partially enriched in fully sampled genes (Fig 8A). As infection progressed, the least accessible chromatin fractions (insoluble and fractions 1 and 2 at 2 h, or insoluble and fractions 1 at 4 or 15 h) were enriched in fully sampled HSV-1 genes of all kinetic classes, as were the most accessible fractions (fractions 11 to 13 at 2 h, or fractions 10 to 13 at 4 or 15 h) (Fig 8A).

Sampling of all genes in all fractions was again averaged and the sampling in each fraction was expressed as enrichment over the average sampling and plotted together with the percentage of HSV-1 DNA in each fraction as in Fig 5B. At 2 h after infection, the least accessible (insoluble chromatin to fraction 2) chromatin that was enriched in fully sampled HSV-1 genes had 72% of the HSV-1 DNA (Fig 8B). The intermediately accessible chromatin that was only partially depleted of fully sampled genes contained 21% HSV-1 DNA (fractions 2 to 10). The most accessible (fractions 11 to 13) chromatin that was only partially enriched in fully sampled HSV-1 genes (20–40% of each gene) had only 7% of the HSV-1 DNA (Fig 8B). The distribution of the viral DNA was somewhat similar to that of the cellular DNA, albeit more enriched in the least accessible chromatin. At 4h, the least (insoluble chromatin to fraction 1) and most (fractions 11 to 13) accessible chromatin that were enriched in fully sampled HSV-1 genes had 57% or 21% of the recovered HSV-1 DNA, respectively. At this time, there was a more marked depletion in gene sampling in the intermediately accessible chromatin, and the distribution of the viral DNA started to more markedly differ from that of the cellular DNA, with a clear enrichment of viral DNA in the most accessible chromatin. At 7 h, as already discussed (Fig 5B–presented again in Fig 8B for comparison), the least (insoluble chromatin to fraction 2) and most (fractions 11 to 13) accessible chromatin that was enriched in fully sampled HSV-1 genes had 35% or 31% HSV-1 DNA, respectively. There was a marked depletion of HSV-1 DNA in the intermediately accessible chromatin and the distribution of cellular and viral DNA differed the most. At 15 h, the least (insoluble chromatin to fraction 1) and most (fractions 10–13) accessible chromatins that were enriched in fully sampled HSV-1 genes had 50% or 30% of HSV-1 DNA, respectively (Fig 8). At this time, HSV-1 DNA was also enriched, together with fully sampled HSV-1 genes, in the intermediately accessible chromatin in fraction 4 (4%).

### Selected short HSV-1 DNA sequences are less underrepresented in the digested and fractionated HSV-1 chromatin than in the undigested and unfractionated one

**Fig 9:**
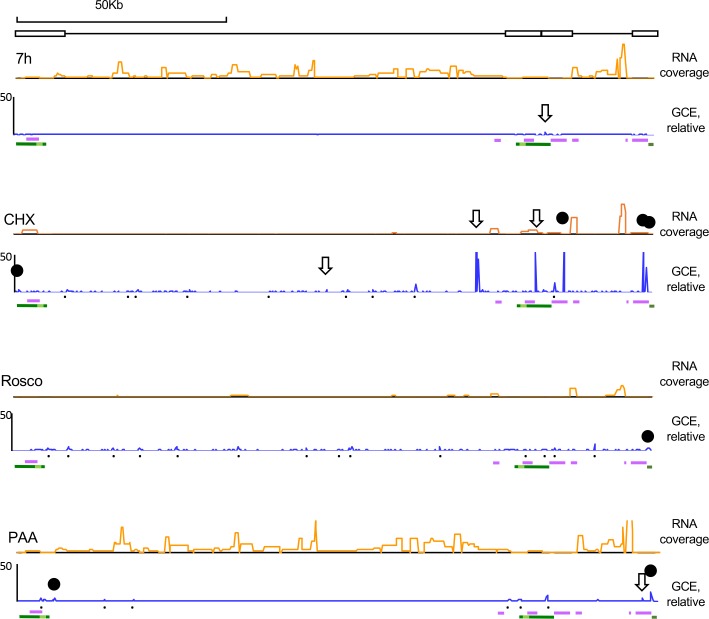
No HSV-1 loci fractionated differently regardless of transcription levels, but short overrepresented sequences flanked transcribed genes when transcription was restricted. Line graphs showing HSV-1 number of genome copy equivalents (GCE) in each genome position in untreated infections, or in infections treated with CHX, Rosco, or PAA at 7 hpi, normalized to the number of HSV-1 genome copy equivalents in the respective position of undigested and unfractionated chromatin (blue line). Orange line graphs, HSV-1 RNA reads. X-axes, genome position (cartoon on top). Black downward empty arrows, CTCF binding sites in strain 17; black solid circles, chromatin insulator-like elements in strain 17; black dots, other overrepresented sequences; purple bars underneath genome plots, IE genes; dark green bars underneath genome plots, LAT; light green bar, stable LAT; GCE, genome copy equivalent. Results from one experiment representative of three.

To analyze if there were large scale differences in the accessibility of different HSV-1 loci, the number of HSV-1 DNA reads for each genome position in each fraction was corrected by the HSV-1 genome copy equivalents in the same fraction. The addition of the genome copy equivalents in each position in all fractions were normalized to the HSV-1 DNA genome copy equivalents in the same position in the unfractionated and undigested chromatin to give the relative genome copy equivalents of that position after digestion and fractionation. The normalized values were plotted against genome position using a 250bp sliding window. The coverage of HSV-1 RNA were plotted likewise to evaluate the transcription levels at each locus (Fig 9).

Consistently with the cluster analyses, the entire HSV-1 genomes were similarly accessible at 7 h, regardless of the transcription level at any specific locus. No genomic region was obviously enriched or depleted in any fractions (S1 Fig), and the total recovery of the HSV-1 DNA in each locus in all fractions was equally proportional to the total HSV-1 DNA in that locus before digestion and fractionation (Fig 9). One sequence in the repeat region appeared to have been relatively enriched to some extent in the digested and fractionated over the undigested and unfractionated chromatin; it mapped to a CTCF binding site [59] (empty downward arrow). As the HSV-1 genome is widely transcribed at 7 h after infection, we analyzed the infections treated with CHX or Rosco to test transcribed and non-transcribed loci.

When E and L gene transcription was inhibited with CHX, only the IE genes were transcribed to a high level (Fig 9), as expected. However, the transcribed IE loci did not fractionate differently from the non-transcribed E or L loci. No obvious HSV-1 genome region, and particularly no IE locus, was enriched in any fraction and compensatory depleted from other (S2 Fig). A few sequencing artifacts map to the same positions in several fractions (fractions 8 through 10, for example) and the undigested and unfractionated chromatin fraction. All HSV loci, including the IE, were also proportionally recovered in the digested and fractionated chromatin over the undigested and unfractionated one (Fig 9). However, 16 short sequences of less than 250bp were relatively overrepresented in the digested and fractionated chromatin over the undigested and unfractionated one (Fig 9). Seven of them, including five of the six most overrepresented, map to previously mapped CTCF binding sites (empty arrows) or chromatin insulator-like elements (black solid circles) in strain 17 [60].

When HSV-1 transcription was inhibited with Rosco, the levels of transcription were low through the entire genome, including the IE genes, as expected, with some low levels of transcription through the genome and lower levels of IE transcription than in CHX treated infections. As in all other conditions, no specific HSV-1 locus was enriched in any fraction and compensatory depleted in others (S3 Fig). The same sequencing artifacts detected in the CHX samples were evident in the same positions (for example, fractions 7 through 10). As in the previous cases, however, 15 short sequences of less than 250bp were slightly and relatively overrepresented in the digested and fractionated chromatin over the undigested and unfractionated one, albeit not nearly as overrepresented as in the CHX-treated infections (Fig 9). One of these sequences map to chromatin insulator-like elements (black solid circle) [59].

To evaluate a condition in which only some loci are not highly transcribed in a context of globally active transcription, we used PAA. No gene loci fractionated differently in infections treated with PAA either, in which HSV-1 IE and E genes were transcribed to high levels whereas the L genes were not. Nine short sequences were again relatively overrepresented in the digested and fractionated chromatin over the undigested and unfractionated one, albeit again not nearly as much as in the CHX-treated infections. Two map to a previously mapped chromatin insulator like elements (black solid circle) [59], and another to a previously mapped CTCF binding site (empty downward arrow) [60].

### The short sequences overrepresented in the chromatin fractions flank highly transcribed HSV-1 genes

**Fig 10:**
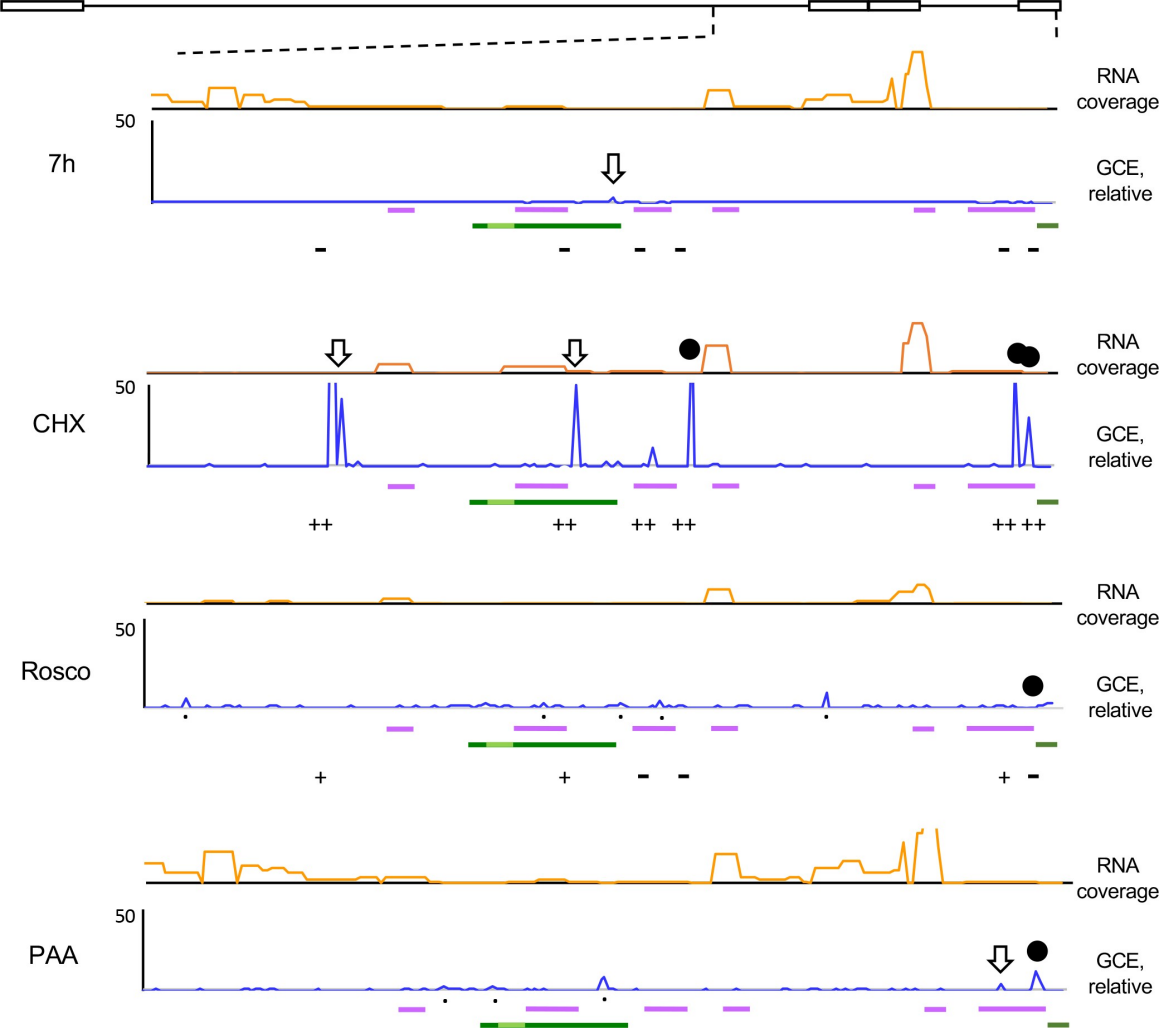
Enlargement of the repeats region, from position 100kbp to 152kbp, of the plots presented in Fig 9. Orange lines graphs, HSV-1 RNA reads. X-axes, genome position (cartoon on top). Blue lines, HSV-1 number of genome copy equivalents (GCE) in each genome position. Black downward empty arrows, CTCF binding sites in strain 17; black solid circles, chromatin insulator-like elements; black dots, other overrepresented short sequences; purple bars underneath genome plots, IE genes; dark green bars underneath genome plots, LAT; light green bar, stable LAT; GCE, genome copy equivalent. Results from one experiment representative of three. ++ sequences co-immunoprecipitated with CTCF by ChIP in two independent experiments; + sequences co-immunoprecipitated with CTCF by ChIP in one of two independent experiments;—sequences not co-immunoprecipitated with CTCF by ChIP in either of the two independent experiments.

As the relatively most overrepresented short sequences in the digested and fractionated chromatin mapped to the internal and terminal repeats, which encode most of the IE genes, the LAT, and some other genes, the HSV-1 repeats were evaluated in more detail (Fig 10). In infections treated with CHX, the six dominant peaks flanked the highly transcribed IE loci, separating them from the rest of the genome (which is non-transcribed, orange line on top). No similar dominant peaks flanking the IE genes were observed in the untreated infections, in which there is abundant transcription across the entire genome, or in the infections treated with Rosco, in which there was little transcription across the genome, or with PAA, in which there is abundant transcription of IE and E but not L genes (Fig 10). No peaks thus separated the IE genes from the rest of the genome when the overall transcription level was high (untreated or PAA treated), or low (infections treated Rosco) across the entire genomes.

The relatively overrepresented sequences were often underrepresented in both the undigested and unfractionated and some also in the digested and fractionated chromatin, which is more obvious when the data are plotted in a logarithmic scale (S4 and S5 Figs). When so, however, they were less underrepresented in the digested and fractionated chromatin than in the undigested and unfractionated chromatin.

There was only one just visible overrepresented short sequence in the digested and fractionated chromatin in the untreated infections, when the overall transcription level was high, nine in PAA treated infections, when L genes are not transcribed, sixteen, including six very predominant ones, in the chromatin of CHX treated infections, when only the IE genes were transcribed, and fifteen in the chromatin of Rosco treated infections, when the overall transcription level was low.

The sequences underrepresented in the undigested and unfractionated chromatin of CHX-treated infections that had not been experimentally mapped as CTCF binding sites in HSV-1 KOS included sequences recognized by CTCF in strain 17 or predicted in silico (S8 Fig) to be potential CTCF binding sites in strain KOS. We thus explored whether these peaks comprised sequences recognized by CTCF. Sequences on the previously described CTRS3, CTRS1/2 and CTa’m [59], as well as the CTCF binding sites recognized by primers B1# and B9# [60] in strain 17, and the proposed new CTCF binding site in the ICP4 gene, all co-immunoprecipitated with CTCF above background in two independent experiments in infections treated with cycloheximide (Fig 10). Consistently with the much reduced magnitude of the overrepresentation of all peaks in the roscovitine-treated infections in the MCN-seq, only the CTRS1/2 sequences and those amplified with primes B1# and B9# co-immunoprecipitated with CTCF above background and each in only one of two independent experiment under these conditions (Fig 10). Consistently with the lack of underrepresented sequences in the undigested and unfractionated chromatin in the MCN-seq experiments, none of these sequences co-immunoprecipitated with CTCF above background in the untreated infections in either of the two independent experiments (Fig 10).

### The number of short sequences that are less depleted in the digested and fractionated chromatin decreases as the infection progresses

**Fig 11:**
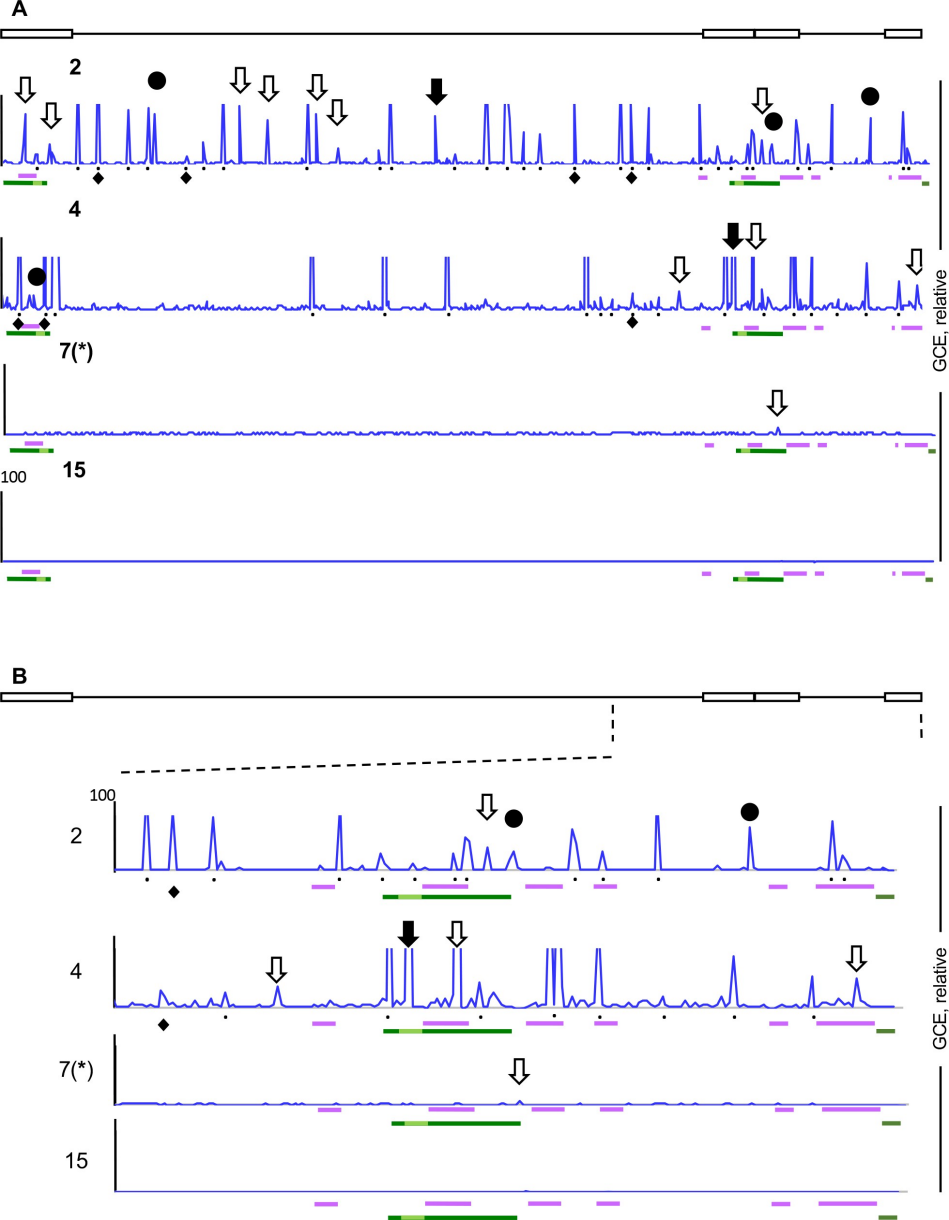
The number of overrepresented short sequences decreases as infection progresses. Line graphs showing number of HSV-1 genome copy equivalents in each genome position at 2, 4, 7 (*), or 15hpi, normalized to the number of HSV-1 genome copy equivalents in the same position of the respective undigested and unfractionated chromatin. X-axes, genome position (cartoon on top). *: data from Fig 9 presented again for comparison. Black downward empty arrows, CTCF binding sites; black solid circles, chromatin insulator-like elements; black downward solid arrows, CTCF binding- and chromatin insulator-like elements; black dots, peaks of the DNA plots; black diamonds underneath the plots, the seven overrepresented sequences that do not contain the AT-rich motifs; purple bars underneath genome plots, IE genes; dark green bars underneath genome plots, LAT; light green bar, stable LAT; GCE, genome copy equivalent. (A) Presentation of relative HSV-1 DNA plots through the entire genome; (B) Enlargement of the relative HSV-1 DNA plots of the repeat region, from position 100kbp to 152kbp. Results from one experiment representative of two.

We next evaluated the number and location of the relatively overrepresented short sequences as the infection progressed. At 2h after infection, 41 short sequences were clearly overrepresented, including 30 predominant ones (Fig 11). Although the total HSV-1 genome copy equivalents were similar to those in the infections treated with CHX, the distribution of these relatively overrepresented sequences was markedly different. The relatively overrepresented short sequences were evenly distributed through the genome at 2 h, rather than clustered flanking the IE genes as in the infections treated with CHX. Nonetheless, most IE genes were still flanked by sequences with limited accessibility (Fig 11B). There were 23, 1 or no short sequences relatively overrepresented at 4, 7 or 15 h, respectively. The overrepresented peaks included, again, previously mapped chromatin-insulator like sequences (solid black circles) [59] and CTCF binding sites (empty downward arrows) [60], or both (black downward arrows) (Fig 11).

## Discussion

Chromatin remodelers are often recruited, via previous histone post-translational modifications, to cellular genes to increase chromatin dynamics and activate transcription [54, 55, 61–64]. One could have expected the histone dynamics on transcribed and non-transcribed individual HSV-1 genes to differ, too. The genes in the more dynamic chromatin would be more accessible to transcription factors and MCN than those in less dynamic chromatin. Under this model, the DNA of IE genes would be more accessible early in infection than that of E and L genes. The HSV-1 genes would therefore fractionate according to their kinetic class after serial MCN digestion and sucrose gradient centrifugation. When analyzing their fractionation patterns, however, all HSV-1 genes clustered together according to the global level of transcription of the genome, regardless of the level of transcription of the individual gene (or kinetic class). Neither the clustering of HSV-1 genes nor the sampling of each gene support the simplest model that chromatin dynamics are the major regulator of the transcription of individual HSV-1 genes. Instead, they rather support an alternate model in which chromatin dynamics regulate the transcriptional competence of entire HSV-1 genomes. As a caveat, populational level approaches preclude the evaluation of differential dynamics of specific genes in small subsets of the viral genomes.

The global accessibility of the HSV-1 DNA differed as infection progressed. At 2h after infection, most of the HSV-1 DNA was in the least accessible chromatin. At this time after infection, the DNA in the promoters of IE (ICP0), E (TK) and L (VP16) genes co-immunoprecipitates with histones bearing silencing markers, and the efficiency of immunoprecipitation of HSV-1 chromatin is comparable to that of cellular chromatin [11, 12, 23, 32, 42, 65, 66], always constrained by the intrinsic limitations of populational analyses and infection synchronization. Recently, Dr. O’Hare’s group used EdC-labeled HSV-1 virions to analyze the nuclear topology of the HSV-1 DNA, enables discriminative analyses of different genomes within an infected cell [67]. As infection progress from 0.5h to 2h, the EdC signal, representing the HSV-1 genomes, became less condensed and more dispersed. These changes are consistent with HSV-1 genomes becoming less compacted, and therefore more accessible, as the infection progresses [67]. Depletion of a chromatin remodeler ATRX also increases mRNA levels of all genes in three HSV-1 kinetic groups at any time of infection up to 8 hours [43]. In our experiments, genes of all three kinetic classes were enriched in the least accessible chromatin at the earlier times (Figs 7 and 8).

As the infection progresses, the efficiency of co-immunoprecipitation of HSV-1 DNA with chromatin decreases, and silencing markers become depleted from the viral chromatin [11, 32]. Overall histone dynamics in the nucleus of infected cells also increase at 4h after infection [7, 68–70]. ChIP of HSV-1 DNA in ATRX depleted cells indicated that histone H3K27me3 occupancy on the ICP27 and ICP8 gene promoters is not significantly affected up to 4 hours of infection, but reduced to about half at 8 hours after infection [43]. In our experiments, HSV-1 DNA started to be enriched in the most accessible chromatin (and perhaps some naked DNA) at this time, too (Figs 7 and 8). Furthermore, DNA from IE, E and L genes mapping to the Ul, Us, and Rs regions of the genome co-immunoprecipitated with histone H3 (Fig 1). These results are consistent with more dynamic interactions of histone H3 with HSV-1 DNA than with cellular DNA.

The increase in histone dynamics and the enrichment in the most accessible HSV-1 chromatin (which may also contain perhaps some naked DNA) could both have resulted from HSV-1 DNA replication. However, PAA has no obvious effects on HSV-1 chromatin. The decrease in efficiency of co-immunoprecipitation of HSV-1 DNA with histones as infection progresses is independent of HSV-1 DNA replication [32], as are the changes in dynamics of histone H1, H2B, H2A, H3.3, and H4 [68–70]. Neither is HSV-1 DNA accessibility to MCN digestion altered by inhibition of HSV-1 DNA replication [6]. Consistently, Dr. O’Hare’s work, using approaches that allow to resolve different genomes in a single cell, showed that inhibition of HSV-1 DNA replication by PAA or acyclovir did not affect HSV-1 genome decompaction as infection progressed [67], and using populational approaches Dr. Knipe’s group showed that PAA does not affect the level of H3K9me3 on HSV-1 chromatin [43].

Several models have been proposed for the potential structures of the HSV-1 chromatin in lytic infections. Only specific regions of HSV-1 genes were protected to nucleosome-sized fragments after complete MCN digestion [13]. Most of the promoters of IE genes, and 60% of the promoters of E genes and L genes, were not detected in the MCN-resistant fraction. The HSV-1 DNA fragments of nucleosome size tended to cluster in the center of the longer genes, whereas short genes and the 5`and 3`ends of most genes were mostly not detected in the DNA subpopulation protected as nucleosome sized fragments [13]. Had these patterns of protection reflected different static levels of nucleosome occupancy, then the serial MCN digestion would have yielded similar protection patterns. The poly-nucleosome fragments of regular HSV-1 chromatin would have resolved to the higher density fractions in the sucrose gradient, whereas the short nucleosome fragments of the partially chromatinized HSV-1 DNA would have resolved to the lighter ones, and the naked DNA would have mostly been digested, or would have resolved to the very top fractions. In contrast, complete HSV-1 genomes partitioned to every fraction, with clear enrichment of HSV-1 DNA in the most and least accessible chromatin (Figs 2D and 5). The patterns of protection from complete MCN digestion [13] are therefore more likely to reflect the dynamics of the HSV-1 chromatin than differential static nucleosome occupancy.

HSV-1 DNA is protected from serial MCN digestion to sizes consistent with nucleosome DNA in complexes that have the same hydrodynamic ratios as mono- to poly-nucleosomes [5, 6] in which it interacts with histone H3 (Fig 1). The most inaccessible chromatin contained 1/3 of the intracellular HSV-1 DNA at 7 hours after infection (Fig 5B). VP5 was barely detectable in the insoluble (or soluble) chromatin at this time. Therefore, most of the DNA in the insoluble chromatin fraction at 7 hours is not likely to be encapsidated. However, ICP4 and ICP8 fractionated to the most inaccessible fractions at this time (Fig 3) and could thus have protected some of the HSV-1 DNA in them. We would not expect to detect much PML at 7 hours after infection. We have not evaluated the fractionation of PML at earlier times or in the presence of cycloheximide or roscovitine and the approaches we have used do not evaluate whether other non-chromatin proteins could have possibly also protected some of the HSV-1 DNA. Although it is thus possible that PML, ICP4, ICP8 or other non-chromatin proteins could have also participated in the protection of the HSV-1 DNA under some conditions, the protected HSV-1 DNA fragments have the same hydrodynamic ratios than mono- to poly-nucleosomes in all tested conditions. Any protecting nucleoprotein complex would thus have to have structures with similar hydrodynamic ratios to actual nucleosomes.

Intracellular HSV-1 DNA pulled down using EdU-labelling and click chemistry was apparently associated mostly with ICP4, VP16 and ICP22 [14]. In our analyses, ICP8 was enriched only in the most accessible chromatin (Fig 3), whereas ICP4 fractionated similarly to HSV-1 DNA, with a shift towards the more inaccessible fractions (Fig 3). ICP4 co-localized with the HSV-1 genomes immediately after infection, but such co-localization was disrupted as the infection progressed. At 3hpi, ICP4 was enriched at the periphery of the replication compartments, where the HSV-1 DNA signal was relatively low [67]. It is possible that EdU-mediated purification enriches for the most dynamic HSV-1 chromatin in which the EdU label would be more easily accessible. Such an enrichment would result in the apparent lack of enrichment in histones, particularly under a stringent enrichment criterion [14, 15] (four-fold increase in the number of the reads over the number in cells infected with non-labelled HSV-1, a very high threshold for most abundant nuclear proteins such as histones). The HSV-1 DNA pulled down by EdU-mediated purification was associated with a number of chromatin remodelers [14], which is consistent with the recovery of mostly HSV-1 DNA chromatinized to a highly dynamic state.

Chromatin epigenetics affect HSV-1 transcription. Inhibition of proteins involved in chromatin epigenetic regulation (Set1, LSD1, Co-REST, JMJD2, or CLOCK complex), by small-molecule inhibitors, knockdown, or knockout inhibits HSV-1 transcription or replication [42, 46, 47, 71]. The HSV-1 transcription activators ICP0, ICP4 and VP16 all interact with chromatin remodelers [19, 20, 29, 47, 50, 51, 72]. ICP0, an E3-ubiquitin ligase, blocks histone deacetylation by interacting with histone deacetylases and the REST/Co-REST complexes [32, 50, 51, 73, 74]. It also induces degradation of silencing histone variants such as Cenp-A and Cenp-B [30, 31]. VP16 recruits histone acetylases, methyltransferases and demethylases to activate HSV-1 transcription [19, 20, 47]. ICP4 increases the dynamics of all core histones [7, 68–70], and interacts with a variety of chromatin remodelers, including the CLOCK complex [29]. All the IE epigenetic regulators that functionally interact with the HSV-1 transcription regulators could very well modulate the dynamics of the HSV-1 chromatin through lytic infections.

Dr. Enquist's group created a pseudorabies virus (PRV) strain containing three fluorescent proteins, each flanked by different recombination sites [75]. Infection of a cre-expressing cell with a single genome of this PRV strain can result in expression of only one randomly recombined fluorescent protein [75]. Infection of a single cell with multiple genomes would be anticipated to result in expression of a mixture of all three fluorescent proteins, each from different randomly recombined genomes. However, it resulted in expression of only one, randomly recombined, fluorescent protein [75]. Moreover, infections with an equal mixture of PRV expressing dTomato, EYFP, or cyan at high moi resulted in most of the infected cells expressing only one of them, or at most two [75, 76]. The simplest mechanism leading to such effects is that only a few viral genomes are biologically active in each infected cell [75, 76]. Recent studies found similar results for HSV-1 [77]. Infection with an equal mixture of 14 HSV-1 different recombinants resulted in an average of only 3.4 or 7.6 of the co-infecting genomes replicated in a given cell at moi of 10 or 100, respectively [77]. The number of replicating genomes correlated with the levels of HSV-1 transcription in each cell, suggesting that HSV-1 transcription and replication competence are regulated together at the genome level. Inhibitors of histone deacetylases (TSA, SBX or VPA) reduced the numbers of transcription competent genomes in each infected cell [78], suggesting that HDACs modulate the transcription competence of the infecting HSV-1 genomes [78]. Histone H3.3 was also enriched in the chromatin of entire HSV-1 genome at 2 or 6h during quiescent infections, without a significant difference between the promoter or the gene body [79]. We show here that some HSV-1 genomes are in most accessible chromatin whereas others are in least accessible chromatin. The accessible genomes were enriched under conditions of ongoing HSV-1 transcription and depleted when HSV-1 transcription was inhibited, which is consistent with the recent results in which the decondensation of the HSV-1 genome was inhibited by restricting HSV-1 transcription using actinomycin D or CHX [67], and the enrichment of silencing histone maker H3K9me3 on HSV-1 chromatin during quiescent infections [79]. It is thus tempting to speculate that the biologically active genomes are in the most accessible chromatin, and the biologically inactive ones, in the least accessible one.

Activation of transcription HSV-1 IE, E or L genes is differentially regulated. The promoters of the IE genes contain multiple transcription factors binding sites, including multiple TAATGARAT, Oct1 and Sp1 binding sites and a TATA box. The promoters of the E genes are simpler. They typically contain only an NF1, a Sp1 binding site, and a TATA box. Those of the L genes are even simpler; they contain only a TATA box. HSV-1 E and L gene promoters are thus likely unable to recruit as many transcription activators or epigenetic modifiers as the promoters of the IE genes do. Therefore, E and L gene promoters may only be able to drive transcription once the IE proteins have disrupted the global silencing of the viral genomes.

Unexpectedly, we found several short sequences that were relatively overrepresented in the MCN-digested and fractionated HSV-1 chromatin, including an overrepresentation of previously described chromatin insulator-like elements and CTCF binding sites. CTCF binding sites and chromatin insulator-like elements function as boundaries between transcriptionally active and inactive genes [59, 80]. CTCF binding was enriched on latent HSV-1 DNA, and CTCF enrichment at CTRL2, CTa'm and CTRS3 was depleted at early times of reactivation [81]. Co-immunoprecipitation of HSV-1 genes with CTCF during lytic infections showed no enrichment of CTCF in CTRL2, CTa’m or CTRS3 clusters [60]. CTCF was instead enriched in the promoters of UL8, UL26 and UL36 genes, on the gene bodies of ICP0 and ICP34.5, and at the 3`of UL25, UL33, UL36 and UL37 genes [60]. Knockdown of CTCF increased H3K9 and H3K27 trimethylation on the promoters and gene bodies of IE (ICP0 and ICP4), E (TK, ICP8) and L (VP16) genes [60], which is consistent with global regulation of HSV-1 genomes by chromatin dynamics. Consistently, studies from Han et al., showed that EGFP expression in absence of any viral immediate early proteins increased when the CMV promoter and the EGFP gene were flanked together by CTRL1 and CTRL2. CTRL1 and CTRL2 decreased the levels of the repressive markers H3K9 and K27 trimethylation in the CMV promoter, but did not alter the levels of repressive markers nor the transcription levels of the neighborhood genes UL51 and UL52 [82]. These studies support role for CTCF in delimiting transcription activity.

The transcribed IE genes (in CHX treated infections) were, interestingly, flanked by the relatively most overrepresented short sequences. Five of these six short sequences mapped to previously experimentally mapped CTCF binding sites or chromatin insulator-like elements in either KOS or 17 (Figs 9 and 10) [59, 60]. As expected, in silico analyses identify at least one potential CTCF binding site in each of these four experimentally mapped binding sites (S8 Fig), and the ChIP assays provided experimental evidence that all six of them are indeed recognized by CTCF in CHX-treated infections (Fig 10). We propose that these sequences with limited accessibility are insulator elements, separating transcribed from non-transcribed genes or genes with vastly different transcription levels. Whether the observed CTCF binding limits the transcription to the IE loci or is a consequence of transcription being limited to these loci remains to be determined.

The short sequences with limited accessibility may also anchor the HSV-1 genome to the nuclear matrix, lamin, or other nuclear structures. In silico analysis of the peaks that did not map to previously mapped (or predicted) CTCF or insulator-like elements (http://meme-suite.org/tools/meme) identified a TA-rich motif (TG/TTTTT/ANA/TA/G in about half of them (25 of 49; S6C Fig). Only fourteen of the peaks in the genomic region encoding most IE genes had neither a CTCF/Insulator or an A/T rich motif. A/T rich motifs are often present in at least a subset of scaffold/matrix attachment regions (S/MARs) [83]. These sequences of limited accessibility are progressively lost as the infection proceeds, perhaps reflecting the lack of genome boundaries when the entire genomes are transcribed or fewer and fewer anchoring points as the infection progresses. Consistently with the latter model, HSV-1 genomes are more compacted when transcription is restricted and genomes decondense as infection progresses [67].

Several of the short sequences relatively enriched in the digested and fractionated chromatin were actually underrepresented in both the undigested and unfractionated and the digested and fractionated chromatins. The relative enrichment of these sequences in the chromatin fractions resulted from their larger underrepresentation in the undigested and unfractionated than in the digested and fractionated chromatin. These sequences are different from the sequencing artifacts, which map to different loci, are conserved in CHX or Rosco-treated infections, and are overrepresented in both in the undigested and unfractionated and digested and fractionated chromatin (S2 and S3 Figs). Chromatin insulators bind to many chromatin insulator binding factors, including CTCF, and interact with multiple nuclear structures to form chromatin loops [84–86]. Like cellular DNA, intracellular HSV-1 DNA also interacts with nuclear structures such as lamin [87]. DNA involved in these anchoring interactions is usually difficult to extract, most likely resulting in its depletion in the unfractionated and undigested DNA. Although nucleases like MCN cannot digest DNA in direct interaction with such structures, they do digest adjacent DNA, thus reducing the size of the DNA trapped in these interactions and therefore decreasing the underrepresentation. The 250bp slide window does not allow for the detection of short missing DNA sequences. The DNA likely trapped in these complex nuclear structures was more depleted in the undigested (and unfractionated) samples than in the MCN digested (and fractionated) ones.

We propose a model whereby chromatin dynamics provides a first level of regulation of HSV-1 gene expression. Cells attempt to silence the infecting HSV-1 genomes by chromatinizing the protein-free infecting HSV-1 DNA to a silenced compacted chromatin. If not all HSV-1 genomes are successfully silenced, then a lytic infection is established. The promoters of the E and L genes are unable to recruit multiple transcription factors to disrupt silencing, whereas those of the IE genes do so via the presence of multiple transcription factors and epigenetic modifiers binding sites. The insulator-like elements anchor the genomes to nuclear structures and prevent transcription from extending to neighboring regions. The viral transcriptional activators, such as ICP0 and ICP4, either as virion proteins or expressed *de novo* in the infected cell, then increase the dynamics of the chromatin in entire HSV-1 genomes, resulting a more relaxed HSV-1 chromatin state and also upregulating E and L gene transcription. Only a few genomes need not be silenced early in infection and become biologically active, while most of the infecting genomes would remain constrained and compacted in silenced chromatin and remain biologically inactive.

## Supporting information

S1 Fig No HSV-1 genomic region was overrepresented in some fractions and compensatively underrepresented in others. Line graphs showing the number of HSV-1 genome copy equivalents (GCE) at each locus in each soluble fraction, the insoluble fraction, the overlap of all insoluble and soluble fractions, and undigested and unfractionated chromatin fraction at 7 hours after infection. Y-axis in logarithmic scale. X-axes, genome position (cartoon on top). Ins, insoluble chromatin fraction.
(TIFF)

S2 Fig No HSV-1 genomic region was overrepresented in some fractions and compensatively underrepresented in others when only the IE genes were transcribed. Line graphs showing the number of HSV-1 genome copy equivalents (GCE) at each locus lots for each soluble fraction, the insoluble fraction, the overlap of all insoluble and soluble fractions, and undigested and unfractionated chromatin fraction at 7 hours after infection treated with CHX. Y-axis in logarithmic scale. X-axes, genome position (cartoon on top). Ins, insoluble chromatin fraction.
(TIFF)

S3 Fig No HSV-1 genomic region was overrepresented in some fractions and compensatively underrepresented in others when HSV-1 transcription was restricted. Line graphs showing the number of HSV-1 genome copy equivalents (GCE) at each locus in each soluble fraction, the insoluble fraction, the overlap of all insoluble and soluble fractions, and undigested and unfractionated chromatin fraction at 7 hours after infection treated with Rosco. Y-axis in logarithmic scale. X-axes, genome position (cartoon on top). Ins, insoluble chromatin fraction.
(TIFF)

S4 Fig The overrepresented peaks result from fewer DNA reads in the unfractionated, undigested HSV-1 DNA in untreated infections or infections treated with Rosco or CHX. Line graphs showing the number of HSV-1 genome copy equivalents (GCE) in each genome position in all fractions (blue) and in the undigested and unfractionated chromatin (black), in untreated infections, or infections treated with CHX or Rosco. Y-axis in logarithmic scale. X-axes, genome position (cartoon on top); upward arrows, the peaks overrepresented in Fig 9; purple bars underneath genome plots, IE genes; dark green bars underneath genome plots, LAT; light green bar, stable LAT.
(TIFF)

S5 Fig The overrepresented peaks result from fewer DNA reads in the unfractionated, undigested HSV-1 DNA as infection progresses. Line graphs showing the number of HSV-1 genome copy equivalents (GCE) in each genome position in all fractions (blue) and in the undigested and unfractionated chromatin (black), in 2, 4, or 15hpi. Y-axis in logarithmic scale. X-axes, genome position (cartoon on top); upward arrows, the peaks overrepresented in Fig 11; purple bars underneath genome plots, IE genes; dark green bars underneath genome plots, LAT; light green bar, stable LAT.
(TIFF)

S6 Fig Many of the short DNA sequences with limited accessibility not previously mapped to CTCF or insulator binding sites contain predicted CTCF binding sites or T/A-rich motifs. (A) Line graphs showing HSV-1 number of genome copy equivalents (GCE) in each genome position in infections treated with CHX, showing two potential CTCF binding sites (CGCCCCCTTGGGGC; GAACTGCC) as predicted by CTCFBSDB 2.0 (http://insulatordb.uthsc.edu/home_new.php). *: these data are from Fig 11 presented again for clarity, as this in silico analysis has no experimental support at this time. (B) Line graphs showing number of HSV-1 genome copy equivalents in each genome position at 2 and 4 hpi showing the 25 potential T/A rich motifs as predicted by MEME (http://meme-suite.org/tools/meme). *: these data are from Fig 10 presented again for clarity, as this in silico analysis has no experimental support at this time. Red bar, a copy of the consensus sequence. (C) The predicted consensus sequence.
(TIFF)

S7 Fig VP5 was below detection levels in any fraction. Western blots of VP5, in the insoluble chromatin and all soluble chromatin fractions after serial MCN digestion and sucrose centrifugation. Results of four independent experiments. The three top blots are over-exposed to better show the lack of signal.
(TIFF)

S8 Fig In silico predicted CTCF binding sites in the six sequences overrepresented in the digested and fractionated chromatin over the undigested and unfractionated one. The line graph from Fig 10 showing the HSV-1 number of genome copy equivalents (GCE) in each genome position in infections treated with CHX, are presented again to show the predicted CTCF binding sites in each of the overrepresented sequences. In silico prediction was performed with CTCFBSDB 2.0 (http://insulatordb.uthsc.edu/home_new.php). *: these data are from Fig 10 and are presented again for clarity, to separate experimental results from in silico predictions.
(TIFF)
