# Supplementary figures and images for: Chromatin dynamics and the transcriptional competence of HSV-1 genomes during lytic infections
Source: PLoS Pathog. 2019 Nov 14;15(11):e1008076. doi: 10.1371/journal.ppat.1008076 (PMC6855408; doi:10.1371/journal.ppat.1008076)

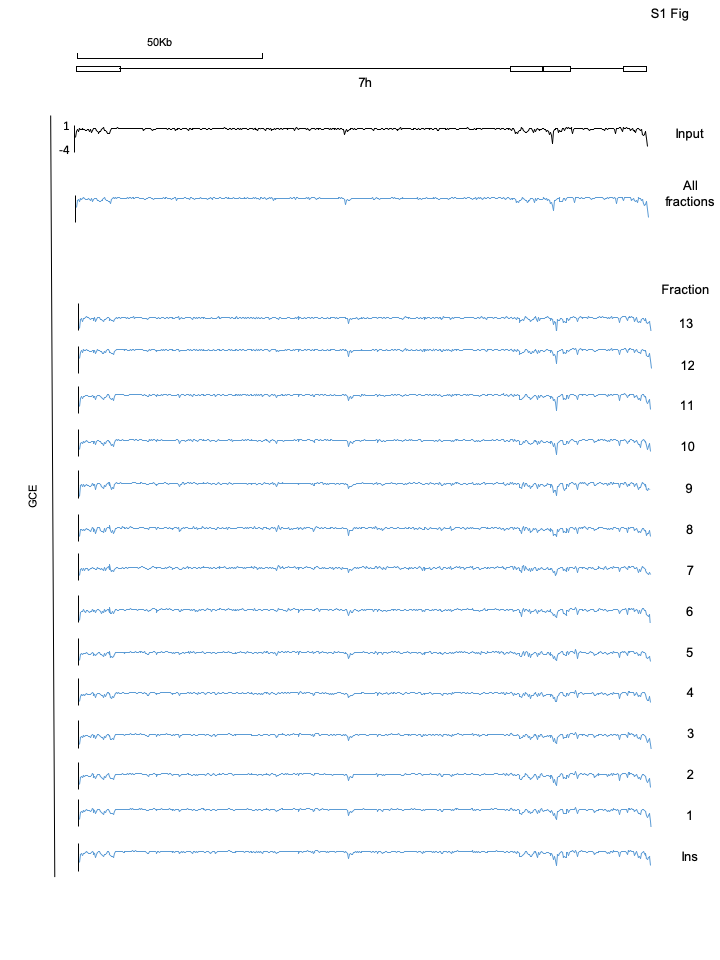

Supplement: S1 Fig — Line graphs showing the number of HSV-1 genome copy equivalents (GCE) at each locus in each soluble fraction, the insoluble fraction, the overlap of all insoluble and soluble fractions, and undigested and unfractionated chromatin fraction at 7 hours after infection. Y-axis in logarithmic scale. X-axes, genome position (cartoon on top). Ins, insoluble chromatin fraction. (TIFF) [file ppat.1008076.s001.tiff]

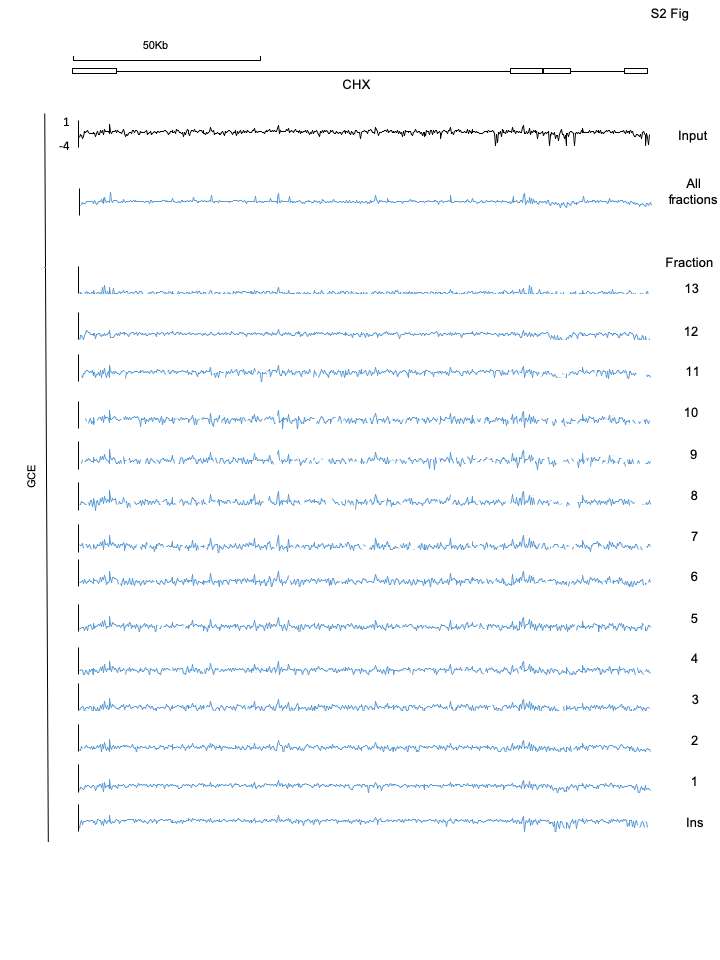

Supplement: S2 Fig — Line graphs showing the number of HSV-1 genome copy equivalents (GCE) at each locus lots for each soluble fraction, the insoluble fraction, the overlap of all insoluble and soluble fractions, and undigested and unfractionated chromatin fraction at 7 hours after infection treated with CHX. Y-axis in logarithmic scale. X-axes, genome position (cartoon on top). Ins, insoluble chromatin fraction. (TIFF) [file ppat.1008076.s002.tiff]

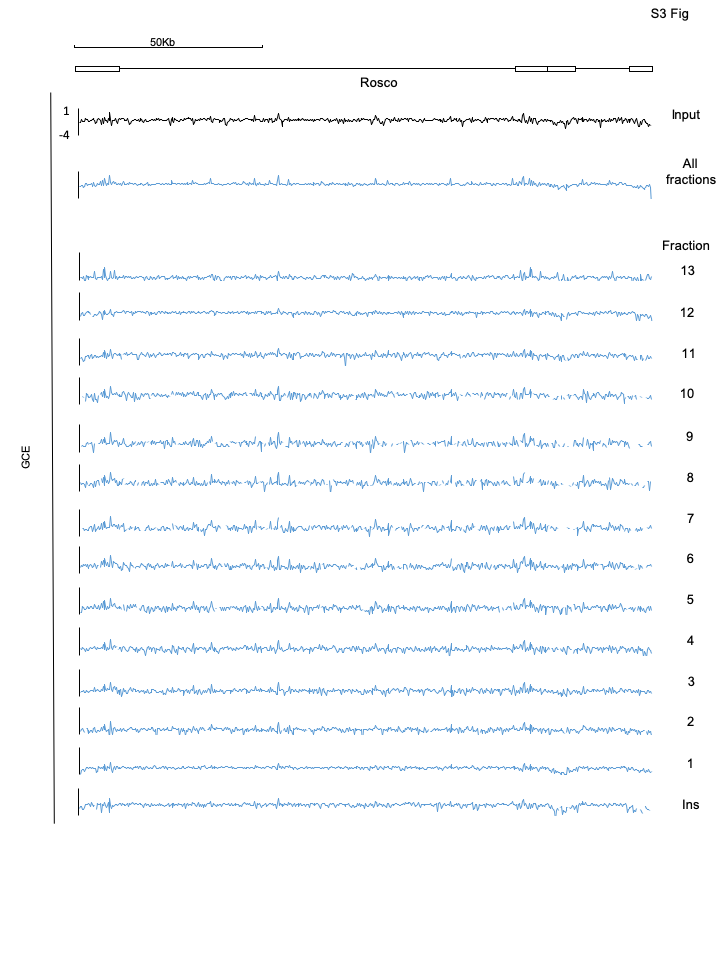

Supplement: S3 Fig — Line graphs showing the number of HSV-1 genome copy equivalents (GCE) at each locus in each soluble fraction, the insoluble fraction, the overlap of all insoluble and soluble fractions, and undigested and unfractionated chromatin fraction at 7 hours after infection treated with Rosco. Y-axis in logarithmic scale. X-axes, genome position (cartoon on top). Ins, insoluble chromatin fraction. (TIFF) [file ppat.1008076.s003.tiff]

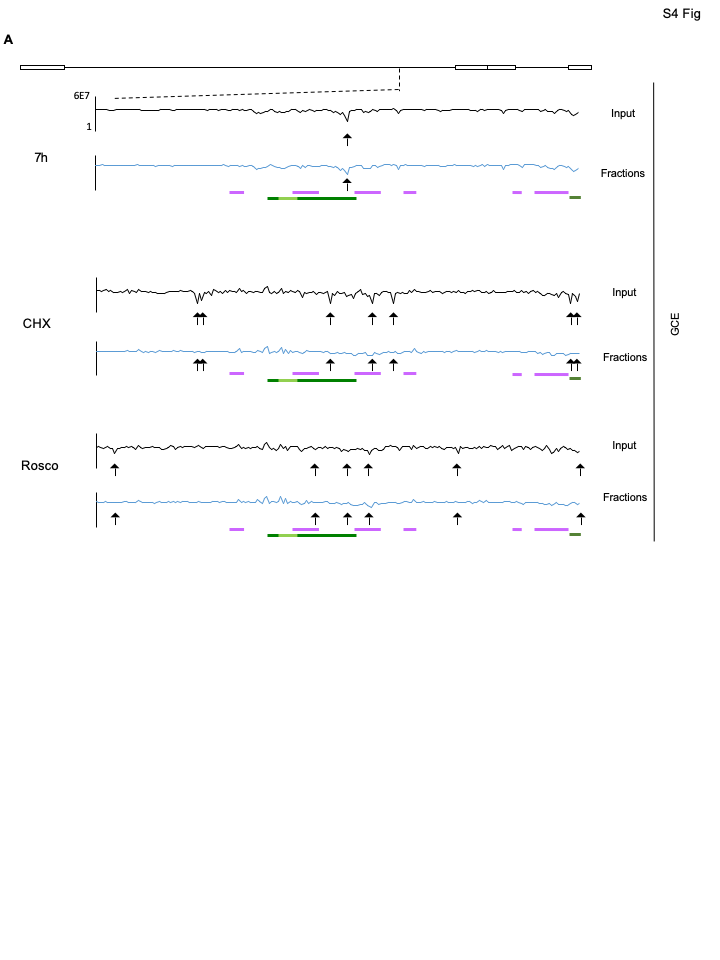

Supplement: S4 Fig — Line graphs showing the number of HSV-1 genome copy equivalents (GCE) in each genome position in all fractions (blue) and in the undigested and unfractionated chromatin (black), in untreated infections, or infections treated with CHX or Rosco. Y-axis in logarithmic scale. X-axes, genome position (cartoon on top); upward arrows, the peaks overrepresented in Fig 9; purple bars underneath genome plots, IE genes; dark green bars underneath genome plots, LAT; light green bar, stable LAT. (TIFF) [file ppat.1008076.s004.tiff]

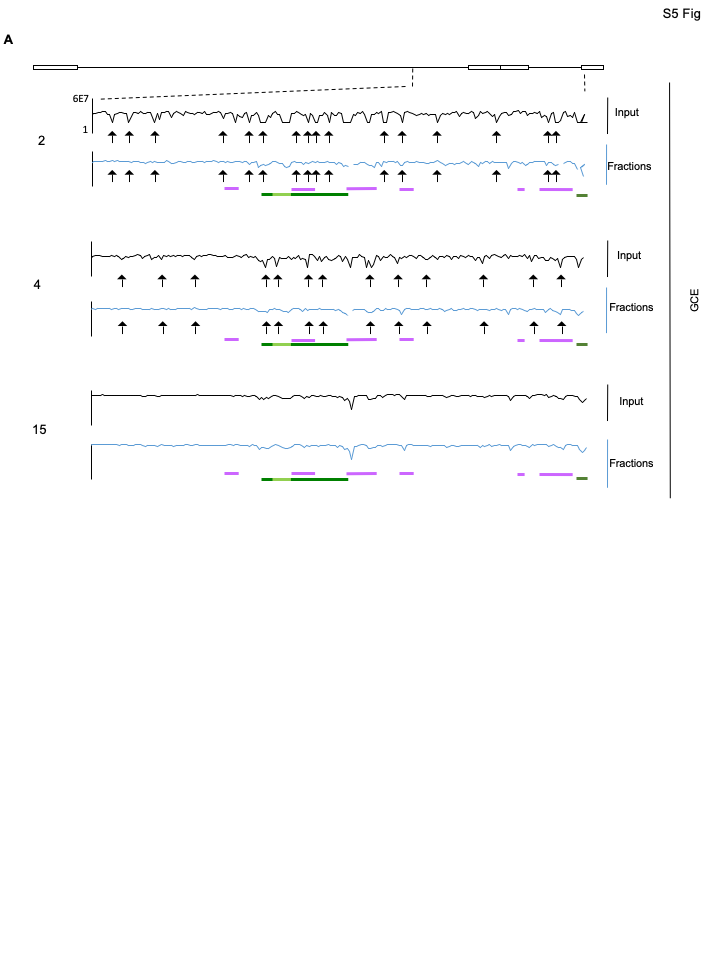

Supplement: S5 Fig — Line graphs showing the number of HSV-1 genome copy equivalents (GCE) in each genome position in all fractions (blue) and in the undigested and unfractionated chromatin (black), in 2, 4, or 15hpi. Y-axis in logarithmic scale. X-axes, genome position (cartoon on top); upward arrows, the peaks overrepresented in Fig 11; purple bars underneath genome plots, IE genes; dark green bars underneath genome plots, LAT; light green bar, stable LAT. (TIFF) [file ppat.1008076.s005.tiff]

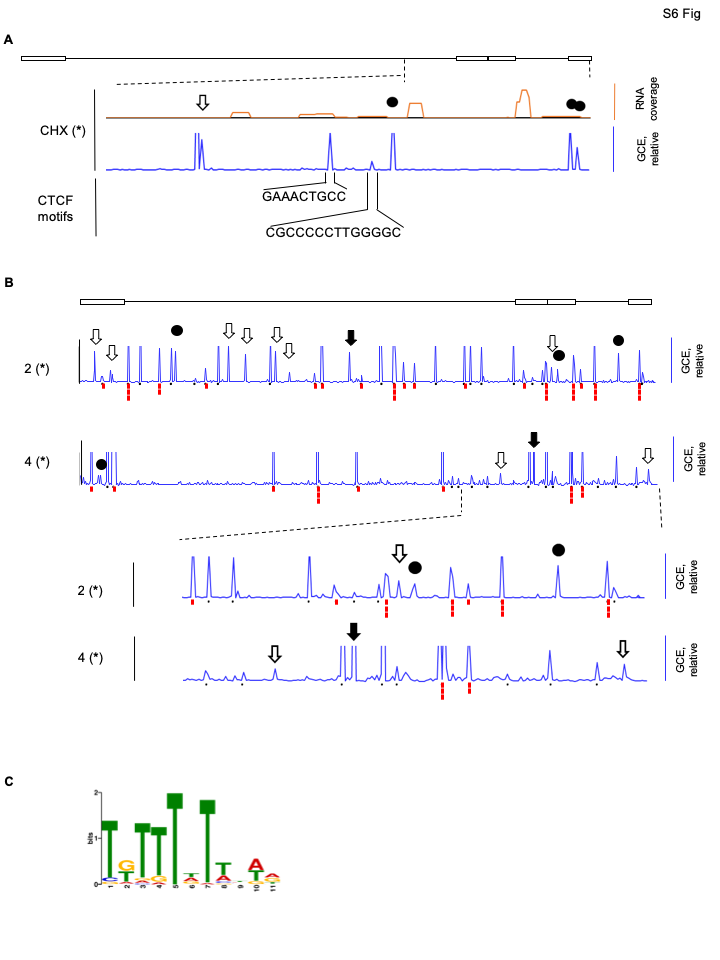

Supplement: S6 Fig — (A) Line graphs showing HSV-1 number of genome copy equivalents (GCE) in each genome position in infections treated with CHX, showing two potential CTCF binding sites (CGCCCCCTTGGGGC; GAACTGCC) as predicted by CTCFBSDB 2.0 (http://insulatordb.uthsc.edu/home_new.php). *: these data are from Fig 11 presented again for clarity, as this in silico analysis has no experimental support at this time. (B) Line graphs showing number of HSV-1 genome copy equivalents in each genome position at 2 and 4 hpi showing the 25 potential T/A rich motifs as predicted by MEME (http://meme-suite.org/tools/meme). *: these data are from Fig 10 presented again for clarity, as this in silico analysis has no experimental support at this time. Red bar, a copy of the consensus sequence. (C) The predicted consensus sequence. (TIFF) [file ppat.1008076.s006.tiff]

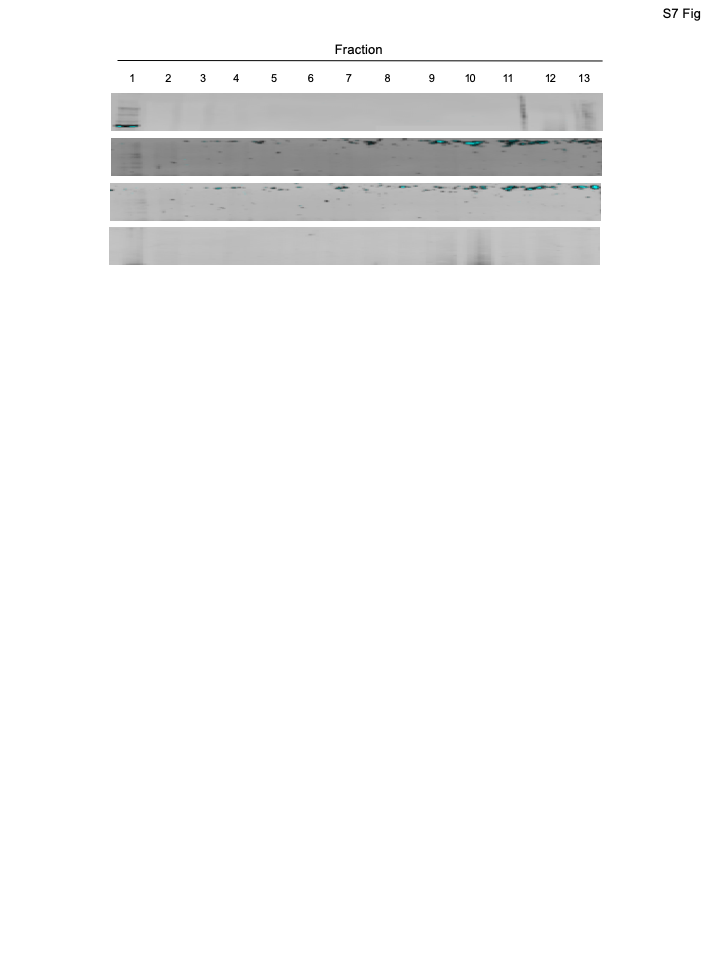

Supplement: S7 Fig — Western blots of VP5, in the insoluble chromatin and all soluble chromatin fractions after serial MCN digestion and sucrose centrifugation. Results of four independent experiments. The three top blots are over-exposed to better show the lack of signal. (TIFF) [file ppat.1008076.s007.tiff]

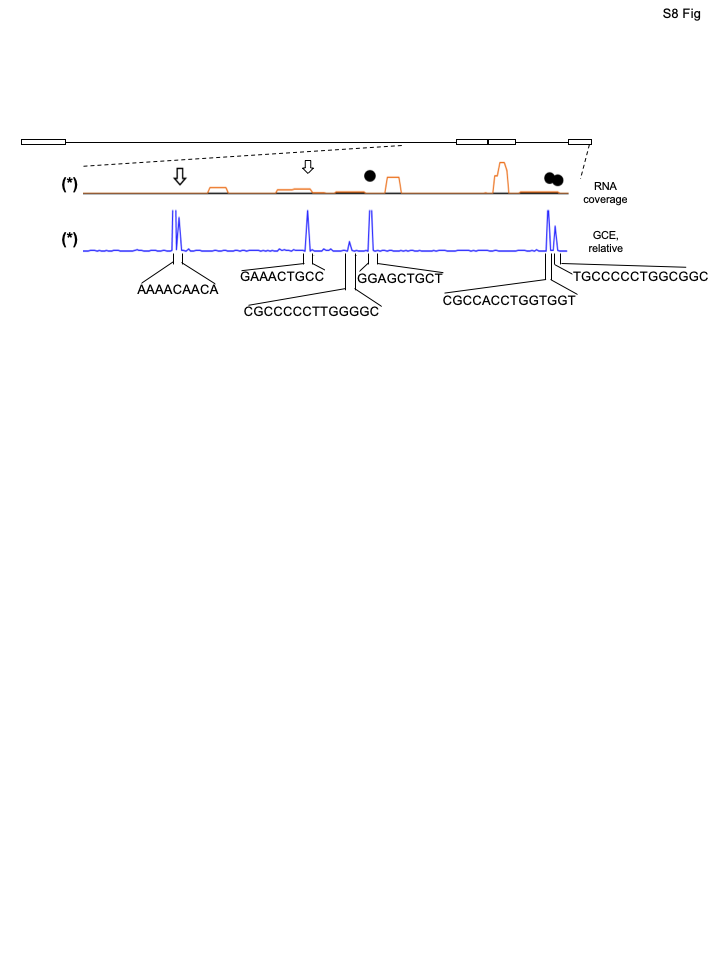

Supplement: S8 Fig — The line graph from Fig 10 showing the HSV-1 number of genome copy equivalents (GCE) in each genome position in infections treated with CHX, are presented again to show the predicted CTCF binding sites in each of the overrepresented sequences. In silico prediction was performed with CTCFBSDB 2.0 (http://insulatordb.uthsc.edu/home_new.php). *: these data are from Fig 10 and are presented again for clarity, to separate experimental results from in silico predictions. (TIFF) [file ppat.1008076.s008.tiff]
